# Finding the adaptive needles in a population‐structured haystack: A case study in a New Zealand mollusc

**DOI:** 10.1111/1365-2656.13692

**Published:** 2022-03-30

**Authors:** Priscila M. Salloum, Anna W. Santure, Shane D. Lavery, Pierre de Villemereuil

**Affiliations:** ^1^ School of Biological Sciences University of Auckland Auckland New Zealand; ^2^ Institute of Marine Science, Leigh Marine Laboratory University of Auckland Warkworth New Zealand; ^3^ Institut de Systématique, Évolution, Biodiversité (ISYEB), École Pratique des Hautes Études PSL, MNHN, CNRS, SU, UA Paris France

**Keywords:** candidate loci, environmental adaptation, gene flow, genome scans for outliers, genotype–environment associations, outlier loci, population genetic structure, q‐values geometric mean

## Abstract

1. Genetic adaptation to future environmental conditions is crucial to help species persist as the climate changes. Genome scans are powerful tools to understand adaptive landscapes, enabling us to correlate genetic diversity with environmental gradients while disentangling neutral from adaptive variation. However, low gene flow can lead to both local adaptation and highly structured populations, and is a major confounding factor for genome scans, resulting in an inflated number of candidate loci.
2. Here, we compared candidate locus detection in a marine mollusc (*Onithochiton neglectus*), taking advantage of a natural geographical contrast in the levels of genetic structure between its populations. *O. neglectus* is endemic to New Zealand and distributed throughout an environmental gradient from the subtropical north to the subantarctic south. Due to a brooding developmental mode, populations tend to be locally isolated. However, adult hitchhiking on rafting kelp increases connectivity among southern populations.
3. We applied two genome scans for outliers (Bayescan and PCAdapt) and two genotype–environment association (GEA) tests (BayeScEnv and RDA). To limit issues with false positives, we combined results using the geometric mean of *q*‐values and performed association tests with random environmental variables. This novel approach is a compromise between stringent and relaxed approaches widely used before, and allowed us to classify candidate loci as low confidence or high confidence.
4. Genome scans for outliers detected a large number of significant outliers in strong and moderately structured populations. No high‐confidence GEA loci were detected in the context of strong population structure. However, 86 high‐confidence loci were associated predominantly with latitudinally varying abiotic factors in the less structured southern populations. This suggests that the degree of connectivity driven by kelp rafting over the southern scale may be insufficient to counteract local adaptation in this species.
5. Our study supports the expectation that genome scans may be prone to errors in highly structured populations. Nonetheless, it also empirically demonstrates that careful statistical controls enable the identification of candidate loci that invite more detailed investigations. Ultimately, genome scans are valuable tools to help guide further research aiming to determine the potential of non‐model species to adapt to future environments.

## Graphical abstract

Reliably interpreting results of genome scans in non‐model species is challenging. Applying genome scans to an understudied brooding chiton and using a new strategy to focus the results on a true signal, this work shows potential for local adaptation even in the face of gene flow.

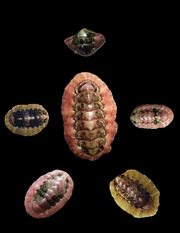

## 1 INTRODUCTION

In the context of climate change, adaptive variation is a critical constraint on a species' distribution, and understanding this variation will help predict a species' potential to respond to future environmental conditions (Razgoura et al., 2019). Currently, the increasing ease of obtaining large panels of genome‐wide molecular markers has been fuelling research on the role of adaptive genetic variation and the identification of genes under selection, even in non‐model species (Nielsen et al., 2009; Pardo‐Diaz et al., 2015).

Genome scans are based on the assumption that natural selection leaves a specific signature in adaptive loci (and in genomic regions in linkage disequilibrium with them) that differs from the genome‐wide variation caused by neutral effects such as genetic drift, gene flow and inbreeding (Beaumont & Balding, 2004; Foll & Gaggiotti, 2008; Nicholson et al., 2002). Such adaptive loci (or their linked markers) are expected to have a distribution of allele or genotype frequencies among populations that do not fit the neutral expectation (Ahrens et al., 2018). The distribution of allele frequencies among populations can also be used as a response variable in tests of association with environmental factors relevant to the ecology of the organism: loci with significant associations are interpreted as being under natural selection due to the focal (or correlated) environmental factors (Ahrens et al., 2018; Bassitta et al., 2021; Dalongeville et al., 2018; Hoban et al., 2016; Liggins et al., 2020; Rellstab et al., 2015; Yadav et al., 2019).

An environment that is highly variable, or structured by a spatial gradient, can subject natural populations to habitat‐specific selective pressures (Kawecki & Ebert, 2004). Permanent variation in these selection pressures across populations may lead to local adaptation via divergent selection (Hedrick, 1986; Hedrick et al., 1976; Sanford & Kelly, 2011). Local adaptation should be more likely when gene flow is lacking among populations in these different habitats. However, recent evidence suggests that local adaptation is not necessarily impeded by gene flow, and the balance between migration and selection also depends on factors such as the strength of selection, and the genetic architecture of traits (Cornwell, 2020; Lenormand, 2002; Pespeni & Palumbi, 2013; Tigano & Friesen, 2016; Yeaman, 2015).

Natural populations with strong population structure are interesting models to understand local adaptation, as they are likely to have low gene flow, and perhaps stronger local adaptation. However, it is known and expected that strong population structure may affect the results of genome scans by increasing the number of false positives (Forester et al., 2018; Frichot et al., 2015; Liggins et al., 2020; Meirmans, 2012). As a consequence, applying genome scans to strongly structured natural populations requires strategies to mitigate this issue.

Here, we exploited a naturally occurring contrast in the strength of population structure in a New Zealand endemic mollusc, *Onithochiton neglectus*, comparing contexts of strong and moderate population divergence, and used a new strategy to combine the results of different genome scan methods. *O. neglectus* is a brooding chiton, lacking a long‐lasting pelagic larval phase and with low‐motile adults (Creese, 1986). With a strikingly broad distribution for its low capacity to move, *O. neglectus* populations are present over a latitudinal gradient of environmental factors from the subtropical northern New Zealand to the subantarctic southern islands (O'Neill, 1985; Salloum et al., 2020). Previously, single‐locus markers (*COI*, *16s* and *ITS‐1*) detected three *O. neglectus* genetic clades, namely the North, Central and South clades (Salloum et al., 2020). Within the North and Central clades, populations are highly differentiated (Salloum et al., 2020). In contrast, within the South clade, *O. neglectus* populations are less genetically structured (Nikula et al., 2012; Salloum et al., 2020). This is likely because connectivity among populations in the South clade is frequently enabled by rafting in the holdfasts of buoyant kelp (*Durvillaea* sp.), which is rarer in the north of *O. neglectus*' distribution (Bussolini & Waters, 2015; Nikula et al., 2012; Salloum et al., 2020; Waters et al., 2018).

**FIGURE 1:**
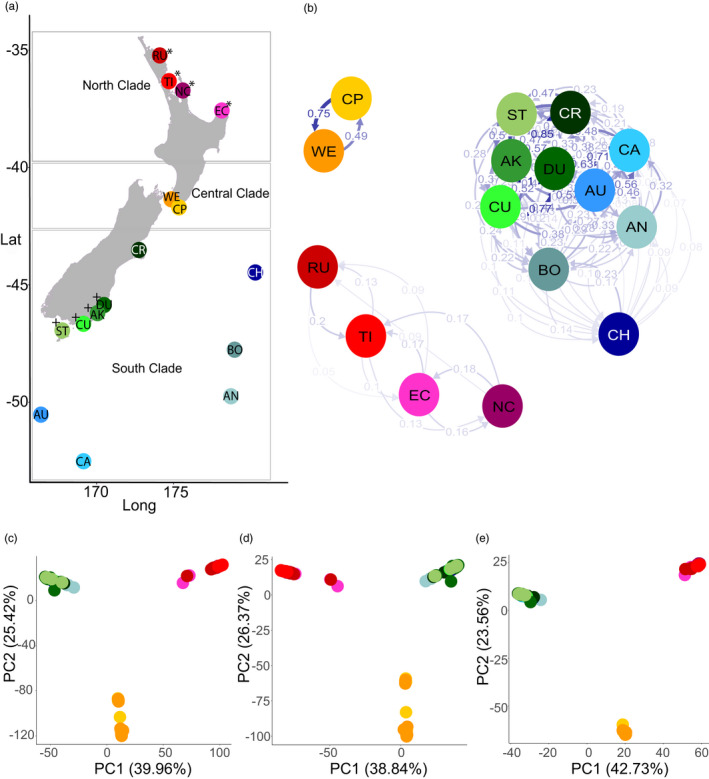
Geographical and genetic relationships among sampled populations of *Onithochiton neglectus*. (a) Geographical location of populations asnd clades. Populations included in the NI dataset are indicated with *, and in the SI dataset with +; (b) relative migration network (estimated using divMigrate). Each circle represents a population (colour‐coded), with the intensity of arrows proportional to migration rate. Rates smaller than 0.05 are not plotted; (c) NZ‐wide PCA for all populations over all loci; (d) NZ‐wide PCA for remaining loci (after removing combined ‘outlier’ markers); (e) NZ‐wide PCA for combined ‘outlier’ markers (combined *q*‐value between PCAdapt and Bayescan smaller than 0.05). Population key: see Table 1

In this study, we used genome‐wide SNPs to identify candidate loci (significant results from genome scans) in this mollusc. We compared four spatial scales: a broad New Zealand‐wide scale with low overall gene flow, including 16 *O. neglectus* populations from three genetic clades (‘NZ‐wide’); a more restricted southern New Zealand regional scale (a subset of nine populations), experiencing higher levels of gene flow due to kelp rafting (‘southern’); a North Island scale, including only the four populations of the North clade (‘NI’); and a South Island scale, comparable with the North Island in geographical range and including only the four South Island populations of the South clade (‘SI’) (Figure 1a; Table 1). We hypothesize that the strong structure of *O. neglectus* populations across a variable environment may lead to populations becoming adapted to local conditions, and expect less evidence of local adaptation among southern populations, which experience higher levels of gene flow and encompass only a subset of the entire environmental variation. Within this framework, we aimed to (a) compare the detection of candidate loci in low versus moderate gene flow scenarios and (b) determine whether population connectivity driven by kelp rafting counteracts local adaptation.

**TABLE 1:** Sampling size per population and their contributions to the associated New Zealand wide (NZ‐wide), upper North Island (NI), South Island (SI) and south and Subantarctic Islands (southern) datasets

Population	Population abbreviation	Individuals	Clade	Dataset
Russell	RU	14	North	NZ‐wide, NI
Auckland	TI	12	North	NZ‐wide, NI
Coromandel	NC	10	North	NZ‐wide, NI
East Cape	EC	9	North	NZ‐wide, NI
Wellington	WE	18	Central	NZ‐wide
Cape Palliser	CP	10	Central	NZ‐wide
Christchurch	CR	6^b^	South	NZ‐wide^a^
Dunedin	DU	20	South	NZ‐wide, southern, SI
Akatore	AK	9	South	NZ‐wide, southern, SI
Curio Bay	CU	5	South	NZ‐wide, southern, SI
Stewart Is.	ST	8	South	NZ‐wide, southern, SI
Auckland Is.	AU	10	South	NZ‐wide, southern
Campbell Is.	CA	10	South	NZ‐wide, southern
Antipodes Is.	AN	7	South	NZ‐wide, southern
Bounty Is.	BO	10	South	NZ‐wide, southern
Chatham Is.	CH	9	South	NZ‐wide, southern

^a^ Population excluded from southern and SI datasets due to high missing data.

^b^ Two additional individuals were sampled but did not meet genomic quality control and were excluded.

To address these aims, we first undertook demographic analyses to explore how variation in the different datasets is structured. Second, we compared geographical patterns of variation from ‘outlier’ sets of markers (derived from significant results of two methods of genome scans for outliers [GSO]: PCAdapt and Bayescan) with those from the remaining loci, to identify potentially different evolutionary histories for these sets of loci. Third, we tested for genotype–environment association (GEA) using two methods (RDA and BayeScEnv) to identify loci correlated with variable environmental factors across the species range (GEA loci hereafter). While false positives can be problematic with genome scans, combining methods can achieve better signal‐to‐noise ratio, at the cost of lower power (de Villemereuil et al., 2014). Thus, we combined the results across the two GSO methods and across the two GEA approaches. To do so, we implemented a novel conservative approach that uses geometric means of *q*‐values between the methods. This aims to balance a mid‐way point between taking all significant results from any method (a union of results, with high power but high false‐positive rate) and only shared significant results across methods (an intersection of results, with low false‐positive rate but low power). Furthermore, to identify GEA loci with a higher probability of reflecting a true association (high‐confidence GEA loci hereafter), after combining *q*‐values across methods we also excluded loci that showed associations with randomly generated variables. Finally, we compared the DNA sequences of high‐confidence GEA loci with a related chiton genome (*Acanthopleura granulata*) (Varney et al., 2021), to identify functional genomic regions.

## 2 MATERIALS AND METHODS

Samples for this study were available from previous work (Nikula et al., 2012; Salloum et al., 2020); thus, no fieldwork was undertaken. *O. neglectus* is not included in the New Zealand Fisheries Act 1996 nor in the New Zealand Wildlife Act 1953; thus, ethical approval was not required.

### 2.1 Sampling, DNA extraction and genotyping‐by‐sequencing

A total of 188 samples of *O. neglectus* from populations distributed across New Zealand and its subantarctic islands were available from previous work (Nikula et al., 2012; Salloum et al., 2020). In all, 16 populations were included in the NZ‐wide dataset (spanning 17 latitudinal degrees, and 167 individuals after filtering, Figure 1a, Table 1, Table S1). A subset of 10 of these populations was included in the southern dataset, representing the previously identified South clade (Salloum et al., 2020). One population (Christchurch) was excluded from the southern dataset due to excess missing genotype data (see below; final southern dataset spanning six latitudinal degrees, and 88 individuals, Table 1, Table S1). The upper North Island (NI) dataset has 45 individuals (two latitudinal degrees), and the South Island (SI) dataset has 43 individuals (one latitudinal degree). Final dataset sizes for each population ranged from 5 to 20 individuals according to how many individuals were collected during previous field sampling (Table 1, Table S1).

DNA extraction, initial checks of concentration and purity were undertaken following the protocol described in Salloum et al. (2020). DNA concentration was normalized and samples were submitted to AgResearch Invermay, Mosgiel, New Zealand, for genotyping‐by‐sequencing (GBS), as detailed in the Supplementary Material (Supporting Information 1). SNPs were called *de‐novo* in Stacks v.2.4 (Catchen et al., 2011; Catchen et al., 2013) for four datasets: New Zealand‐wide across all populations (NZ‐wide), within the North Island (NI) and South Island (SI), and within the South clade (southern). The Central clade was not called separately as it had only two populations, and the North clade is the same as the NI dataset. SNPs with minor allele frequency <0.05 were filtered out. To exclude loci in strong linkage disequilibrium (LD), one SNP per rad‐tag was chosen at random, decreasing the possibility of having SNPs across loci in LD. Finally, individuals with more than 90% missing data (two individuals from the Christchurch population) and rad‐tags with more than 30% missing data were removed (full details of software and parameter settings are provided in Supporting Information 1, Figures S1–S3).

### 2.2 Demographic patterns

Population differentiation and genomic diversity (observed and expected heterozygosity, overall *F*_ST_, and Nei's *F*_IS_) were estimated for all datasets, with the r package hierfstat v. 0.5‐7 (Goudet & Jombart, 2020). The same package was used to estimate overall observed heterozygosity within each population in each dataset, and to check heterozygosity distribution across loci within populations. Principal component analyses were done with the r package lea v. 2.6.0 (Frichot & François, 2015) for all datasets (Table S2). Allele frequencies were compared for all datasets with a custom R script. In addition, Weir and Cockerham's *F*_ST_ was estimated for all loci with VCFtools (Danecek et al., 2011) for pairwise comparisons between clades in the NZ‐wide dataset (North–South, North‐Central and Central‐South). The allele frequency spectrum was derived for each population NZ‐wide, using vcf2sfs (Liu, 2020). In addition, δaδi (Gutenkunst et al., 2009) was used to plot 2D allele frequency spectra between North‐Central, North–South and Central‐South clades. To further analyse population structure in the NZ‐wide dataset, a population assignment plot was done with the r package Adegenet v. 2.1.1 (Jombart, 2008; Jombart & Ahmed, 2011), as this enables the optimal number of ancestral populations and the admixture proportions of these different ancestries within each individual to be inferred (Jombart & Ahmed, 2011). Finally, directional relative migration was estimated for the NZ‐wide dataset with the DivMigrate function in the DiveRsity r package (Keenan et al., 2013), using the *G*_ST_ method (Sundqvist et al., 2016). The relative migration rates were then used in a Pearson's correlation test with a matrix of geographical distance between populations (shortest straight distance, in kilometres, between populations calculated using https://www.distance.to, Table S3).

### 2.3 Genome scans for outliers

To identify ‘outlier’ markers, two GSO were run on each dataset separately, the PCA‐based method PCAdapt (Luu et al., 2017) and the Bayesian method Bayescan (Foll & Gaggiotti, 2008). For PCAdapt, principal component analyses were initially undertaken for 50 principal components (20 for the NI and SI datasets), with the default 5% threshold for minor allele frequencies and no LD thinning (LD was controlled by the random choice of one SNP per rad‐tag when calling SNPs). The default Mahalanobis distance was used as a test statistic, and *p*‐values were corrected using the genomic inflation factor. The proportion of variance explained by the principal components was checked (Figure S4) and the analysis was re‐run with three principal components for the NZ‐wide dataset, two principal components for the NI dataset, one for the SI dataset, and six for the southern dataset, using the same settings as above. After checking their distribution (Figures S5–S11), the corrected *p*‐values were transformed into *q*‐values with the qvalue package v. 2.16 in r (Storey et al., 2019). For Bayescan, runs were performed with the default configurations (5,000 iterations, thinning interval = 10, 20 pilot runs of 5,000 length, burn‐in = 50,000, prior odds for the neutral model = 10).

The results of both methods were combined by calculating geometric means of the *q*‐values of PCAdapt and Bayescan for each SNP, that is, ${\text{qval}}_{\text{combined}}=\sqrt[{2}]{{\text{qval}}_{A}\times {\text{qval}}_{B}},$ where *qval*_A_ and *qval*_B_ correspond to the locus *q*‐value resulting from the two different methods. The geometric mean can be seen as a form of arithmetic mean on the logarithmic scale and, compared to the absolute scale, is therefore more closely linked to the natural interpretation of the *q*‐value. Furthermore, for quantities such as *q*‐values, the geometric mean is less sensitive to extreme values compared to the arithmetic and harmonic means. By combining *q*‐values in this way, a SNP does not need to be significant in both methods, and strong support from one method (a small *q*‐value) can outweigh no support from the other method (a large *q*‐value). For example, a locus with *q*‐value = 0.001 in one method and 0.2 in another has a combined *q*‐value (by the geometric mean) of 0.01, which is significant for a threshold (alpha) of 0.05. This approach recognizes that different methods have different assumptions and may return complementary results, but also reduces the effect of false positives from taking the union of significant associations, while being less stringent than the common practice of taking the intersection of methods (Figure S12).

‘Outlier’ markers with a combined *q*‐value smaller than an arbitrarily chosen 0.05 cut‐off were checked for observed heterozygosity and missing data across populations. Past demographic patterns were inferred for the combined ‘outlier’ markers and for the remaining loci (i.e. having removed combined ‘outlier’ markers from the dataset) in the NZ‐wide dataset with the program Stairway Plot 2 (Liu & Fu, 2020), as well as for each clade separately. A plot of allele frequencies, a PCA and a plot of the distribution of overall *F*_ST_ were done with the combined ‘outlier’ markers and the remaining loci for each dataset. For comparison with the NI, SI and southern datasets (where SNPs were called independently within these groups), the genome scans, PCAs and allele frequency plots were also done for these groups by subsampling the NZ‐wide SNP dataset into the North Island, South Island and southern regions (Table S2).

### 2.4 Genotype–environment association

The PCA‐based method RDA and the Bayesian method BayeScEnv were used to search for loci in association with environmental variables (GEA‐loci). Environmental variables (described below) were mean‐centred and scaled to a variance of one. RDA implemented in the r package vegan v. 2.5.6 (Oksanen et al., 2019) does not allow missing data; therefore, missing loci were imputed (imputing with the most common genotype within the clade). In addition, the environmental variables used as predictors in the RDA should not be correlated (Dormann et al., 2013); thus, only variables with a squared correlation coefficient smaller than or equal to 0.7 with each other were included, leaving six semi‐independent variables in the NZ‐wide and southern datasets, and three semi‐independent variables in the NI and SI datasets (Tables S4 and S5). The RDA performed was set to scale SNP loadings to unit variance. Analyses of variance (ANOVA) with 999 permutations were undertaken to test the RDA full model for significance. To obtain *q*‐values, RDA loadings treated as *z‐*scores were mean‐centred and scaled to unit variance, which were transformed into two‐tailed *p*‐values that were converted to *q*‐values using the qvalue r package (Storey et al., 2019), following checks of their distributions (Figures S13–S16).

For BayeScEnv (de Villemereuil & Gaggiotti, 2015), correlation between environmental variables is not of concern because they are tested independently in multiple runs of a univariate method; thus, all environmental variables were included. In addition, no imputation is required for BayeScEnv analyses. Runs were performed with the default configurations (5,000 iterations, thinning interval = 10, 20 pilot runs of 5,000 length, burn‐in = 50,000, upper bound for the Uniform prior of parameter g = 10, mean alpha prior = −1, prior probability for non‐neutral models = 0.1, prior preference for the locus‐specific model = 0.5) in the NZ‐wide, NI, SI and southern datasets.

The environmental variables available to test in the GEA analyses were latitude, longitude and long‐term means of monthly sea‐level pressure (Kanamitsu et al., 2002), net shortwave, precipitation rate, surface pressure, total cloud cover, zonal wind velocity, meridional wind velocity, air temperature (Kalnay et al., 1996), ocean temperature, ocean salinity (Monterey & Levitus, 1997), salinity, sea surface height relative to geoid (Behringer & Xue, 2004) and sea surface temperature (Ishii et al., 2005). With the exception of latitude and longitude, environmental variables were downloaded from NOAA (data provided by the NOAA ESRL Physical Sciences Division, Boulder, Colorado, USA, from their website at http://www.esrl.noaa.gov/psd/) as netcdf files, and converted to a data frame with the ncdf4 v. 1.16.1 r package (Pierce, 2019). The geographical coordinates of the populations sampled and of the points where environmental data are available are not exactly matched in NOAA's satellite data; thus, the value of the closest point to the actual sample coordinates was used.

In addition, RDA and BayeScEnv were also run using a set of 100 randomly generated variables, normally distributed with a mean of zero and standard deviation of one. Our rationale behind these runs was to identify loci that generated spurious genotype–environment association, suggesting that significant results with the true environmental data for these loci are more likely to be spurious association. Both methods were run using the same parameters as for the real environmental variables.

The results of the two GEA methods were combined using the same method as for GSO: calculating geometric means of the *q*‐values of RDA and BayeScEnv for each locus and environmental variable pair, in the real and random datasets. Loci identified as significantly associated with real variables (combined *q*‐value <0.05) were then examined for association with the randomly generated variables. Those loci that also significantly associated with fewer than five random variables (i.e. 5% of the random runs) were treated as high‐confidence associations. Since BayeScEnv runs included the environmental variables correlated with latitude (*r* ^2^ > 0.7), *q*‐values of all latitude‐correlated variables were combined using the geometric mean, and significant associations to latitude in the ‘broad sense’ are reported. High‐confidence GEA loci were checked for observed heterozygosity and missing data across populations.

Sequences of high‐confidence GEA loci were submitted in a BLAST search against a custom database containing the *Acanthopleura granulata* genome and transcriptome (Varney et al., 2021), using the blastn algorithm with default configurations (Altschul et al., 1990). To identify genes and functions, the longer *A. granulata* transcriptome sequences matching our short *O. neglectus* queries (92‐base fragments) were extracted and run in another BLAST search against other molluscs (taxid:6447) using the nucleotide (nr/nt) database, and the program selection blastn, optimizing for somewhat dissimilar sequences.

## 3 RESULTS

### 3.1 SNP datasets

The SNP calling pipeline resulted in a NZ‐wide dataset consisting of all 16 populations and 10,987 loci, with population‐average 0.2% missing data (Figure S3A, Table 1, Table S2). SNPs were called with a population map grouping the three clades because it resulted in a more complete dataset with less missing data than grouping by population only (average 11% missing data; Figure S3B). The NI dataset consists of 12,012 loci with 0.0% missing data (Figure S3C, Table 1, Table S2). The SI dataset consists of 7,476 loci with population‐average 0.1% missing data (Figure S3D, Table 1, Table S2). The southern dataset consists of 9 populations and 13,004 loci, with population‐average 8.7% missing data (Figure S3E, Table 1, Table S2). One population (Christchurch; CR) was removed from the SI and southern datasets due to excess missing data; note that it had less missing data in the NZ‐wide dataset and hence is included there.

### 3.2 Demographic patterns

All population differentiation and genomic diversity analyses support the subdivision of *O. neglectus* into the three genetic clades previously identified by single‐locus mtDNA and nDNA analyses (Salloum et al., 2020): North, Central and South. Sampled populations within the South clade (in SI and southern datasets) show much less genetic differentiation than that shown among populations within the other clades (Table 2). The same pattern is recovered by all analyses of population structure (gene flow: Figure 1b; PCA: Figure 1c, Figures S17–S23; assignment plots: Figure S24), and allele frequencies NZ‐wide show loci differentially fixed between clades (Figure S17). In addition, the SNP datasets subsampled from the NZ‐wide dataset (NI, SI and southern subsets) show very similar patterns to those datasets with SNPs called separately (NI, SI and southern specific), although the latter all have a larger number of loci (Figures S18–S23, Table S2). Distributions of pairwise *F*_ST_ between clades show strong differentiation between each of the three clades with high *F*_ST_ (North‐Central = 0.713; North–South = 0.639; Central‐South = 0.643, Figure S25). The allele frequency spectrum plots reveal a broadly different pattern for all populations in the South clade as compared to populations from other clades, as southern populations have a higher proportion of low‐frequency alleles (Figure S26). Note that the population allele frequency spectra are based on counts from the NZ‐wide datasets, and the larger number of South populations means the south alleles are the majority, and thus taken as reference. The 2D allele frequency spectra between clades show no correlation pattern, with most of the high‐count alleles in one clade missing or infrequent in the other, indicating no evidence of introgression (Figure S27). The distribution of observed heterozygosity per population NZ‐wide shows most loci with low heterozygosity (below 0.2, Figure S28, Table S6). Finally, relative migration estimates show much higher connectivity among populations of the South clade than among populations of other clades, with no support for migration between clades (Figure 1b, Table S7). Relative migration rates and   geographical distances between populations are negatively correlated (*R* = −0.41, *p* < 0.001, Figure S29, Tables S3 and S7).

**TABLE 2:** Overall genetic diversity and population differentiation (among all sampled populations and using all loci in each specific dataset). No. pops, number of populations included in the dataset; no. Inds, number of individuals included in the dataset; ho, observed heterozygosity; he, expected heterozygosity; Fst, *F*_ST_ based on gene diversity (gene diversity among samples/overall gene diversity); Fis, *F*_IS_ following Nei, 1987 (= 1 − Ho/Hs)

Dataset	No. pops	No. Inds	Ho	He	Fst	Fis
NZ‐wide	16	167	0.057	0.062	0.792	0.083
NI‐specific	4	45	0.198	0.206	0.308	0.041
SI‐specific	4	43	0.255	0.277	0.048	0.079
Southern specific	9	88	0.218	0.232	0.165	0.060

### 3.3 Genome scans for outliers

**FIGURE 2:**
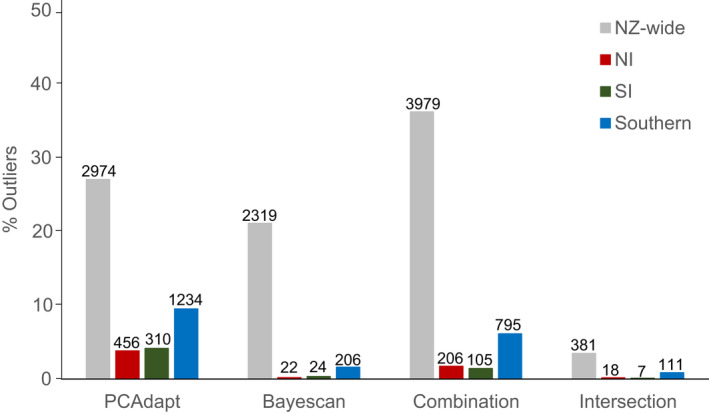
Proportion of ‘outlier’ markers found with each method in the NZ‐wide, NI‐, SI‐ and southern‐specific datasets. Combination represents the number of ‘outlier’ markers after combining the *q*‐values of PCAdapt and Bayescan using the geometric mean; intersection represents the number of ‘outlier’ markers found in common between PCAdapt and Bayescan

PCAdapt identified 27% of the 10,987 NZ‐wide loci as ‘outlier’ markers, 3.8% of the 12,012 loci in the NI‐specific dataset, 4.1% of the 7,476 loci in the SI‐specific dataset and 9% of the 13,004 loci in the southern‐specific dataset (Figure 2). In comparison, Bayescan identified 21% of the NZ‐wide loci as ‘outlier’ markers, 0.2% of the loci in the NI dataset, 0.3% of the loci in the SI dataset and 1% in the southern dataset (Figure 2). There was a much smaller number of ‘outlier’ markers found to be in common (intersection) between the two genome scan methods (Figure 2). Combining the two methods using the geometric mean of *q*‐values resulted in a larger number of ‘outlier’ markers NZ‐wide than each of the methods separately, and in intermediate numbers in the other datasets (Figure 2). Of the 3,979 combined ‘outlier markers’ in the NZ‐wide dataset, 35 had observed heterozygosity above 0.5. In addition, 73 were completely missing in a population and were removed from downstream analyses. In the southern dataset, out of the 795 combined ‘outlier’ markers, 290 were completely missing in one population, and 247 were missing from two or three populations, and all these were removed from downstream analyses. No ‘outlier’ marker was completely missing from populations of the NI‐ and the SI‐specific datasets. The allele frequency spectra of the ‘outlier’ markers are broadly similar to that of the remaining loci for all populations (i.e. the set of loci after ‘outlier’ markers are removed), but often have a greater proportion of high‐frequency alleles (Figure S26). The stairway plots show a recent bottleneck (between one and five thousand years ago) in both ‘outlier’ markers and the remaining loci, but the former have a   larger final effective population size (Figure S30). The general pattern for each North, Central and South clades does not differ among different sets of loci.

The NZ‐wide population structure derived from all SNP loci (Figure 1c) is similar to the structure shown by combined ‘outlier’ markers and by the remaining loci (Figure 1d,e), all strongly supporting the three genetic clades previously described (Salloum et al., 2020). The patterns of ‘outlier’ markers and the remaining loci are also broadly similar in all spatial scales (NI, SI and southern, Figures S17–S23), but it is clear that the remaining loci exhibit relatively more differentiation among nearby populations, while ‘outlier’ markers may reflect higher *F*_ST_ overall (Figures S17–S23 and S31). The ‘outlier’ markers show lower heterozygosity than the remaining loci in most populations of all datasets, except for populations of the South clade in the NZ‐wide dataset (Table S6).

### 3.4 Genotype–environment association

Many of the environmental variables tested are strongly correlated with latitude at all scales (NZ‐wide, NI, SI and southern, Figure S32). The exceptions are precipitation rate, surface pressure, sea surface height relative to geoid and longitude, which are only weakly correlated with latitude. The RDA full model indicates a significant relationship between loci and the associated environmental variables (NZ‐wide scale RDA full model *F*_(5,161)_ = 27.05, *p*‐value < 0.001, NI scale RDA full model *F*_(3,41)_ = 5.95, *p* < 0.001, SI scale RDA full model *F*_(3,39)_ = 1.65, *p* < 0.001, the southern scale RDA full model *F*_(5,82)_ = 3.69, *p* < 0.001).

The RDA analyses identified no genotype–environment associations in the NZ‐wide, NI and SI datasets, but 708 associations in the southern dataset (5% of the loci). In comparison, BayeScEnv identified 3,093 associations from the New Zealand‐wide dataset (28% of the loci), 15 from the NI dataset (0.13% of the loci), 10 from the SI dataset (0.13% of the loci) and 135 from the southern dataset (1% of the loci) (Table 3, Tables S4 and S5). After combining *q*‐values between RDA and BayeScEnv, and removing loci associated with random variables, no high‐confidence GEA loci remained at the NZ‐wide, NI and SI scales, but 86 GEA loci remained at the southern scale, corresponding to 0.6% of the loci (Table 3). Of these, 81 are associated with latitude in the broad sense, three with longitude, one with precipitation rate, and one with both longitude and sea surface height relative to geoid (Table S4). Within the 86 high‐confidence GEA loci, one had observed heterozygosity above 0.5, 33 were found to be completely missing from one population, and 41 from two or three populations, and were not included in the BLAST searches.

**TABLE 3:** Number of GEA loci for each spatial scale and statistical treatment. ‘Real variables’ refer to the total number of associations with environmental variables; ‘5% random variables’ is the number of associations with at least 5% of the random variables (note that these loci are not necessarily also associated with environmental variables); ‘combined *q*‐value’ is the number of significant GEA loci after combining the *q*‐values of RDA and BayeScEnv using the geometric mean; ‘Final High‐confidence loci’ are GEA loci with combined *q*‐value <0.05 and not associated with >5% of the random variables. The total number of loci in each dataset is shown within parentheses

Environmental dataset	Statistical treatment	NZ‐wide (10,987)	NI (12,012)	SI (7,476)	Southern (13,003)
Real variables	RDA	0	0	0	708
	BayeScEnv	3,093	15	10	135
	Combined *q*‐value	0	0	0	171
5% random variables	RDA	0	0	0	693
	BayeScEnv	722	9	19	139
	Combined *q*‐value	0	2	0	90
**Final**	**High confidence**	**0**	**0**	**0**	**86**

BLAST analyses of the sequences flanking the high‐confidence SNPs against *A. granulata* scaffolds returned two matches, one of which also matched the *A. granulata* transcriptome (Table S8). These two *O. neglectus* loci (#14183 and #46591) are associated with latitude. BLAST analysis of the *A. granulata* sequence flanking these matches did not return annotated genes in other molluscs.

## 4 DISCUSSION

To study the genetic basis of local adaptation in a mollusc species in the context of different geographical scales and population structure, we leveraged a natural contrast in the strength of *O. neglectus* population divergence by comparing patterns for all populations (NZ‐wide, with strong population structure) and NI populations (moderate population structure) with patterns in the SI and southern scales (showing lowest population structure). Our overall results agree with the expected artefactual inflation in the number of candidate loci under strong background population structure (Ahrens et al., 2018; de Villemereuil et al., 2014; Excoffier et al., 2009; Forester et al., 2016; Frichot et al., 2015; Hoban et al., 2016; Liggins et al., 2020; Meirmans, 2012; Storfer et al., 2018), although other factors known to influence genome scans cannot be ruled out (see below). For three of the four methods employed, the total proportion of candidate loci detected NZ‐wide is much larger than within the other scales. Considering that 2%–10% of the loci in a dataset are usually found to be significant in this type of analyses (Bierne et al., 2011), our numbers of NZ‐wide candidate loci are extremely high and are likely to include many false positives. However, our GEA analyses revealed 86 high‐confidence loci (with an increased probability of being true positives) within the southern region. Thus, despite greater gene flow in this region, there are candidate loci suggesting potential for local adaptation.

Apart from population structure, there are other factors that can lead to an increase in false‐positive rates of genome scans (GSO and GEA). Not all loci in the genome will follow the same evolutionary path, which results in some loci that are more divergent among populations than others (Roux et al., 2016). The higher divergence in such loci is not necessarily caused by non‐neutral factors (Roux et al., 2016). For example, variation in mutation rates can produce genomic regions of low variability and hence mimic footprints of selection (Thornton & Jensen, 2007). Recent demographic events such as bottlenecks can lead to unusual genealogies for different loci so that they coalesce at different times in the past (Hermisson, 2009; Thornton & Jensen, 2007). Recent research highlights the impact of differences in recombination rate across the genome, which tends to be lower near centromeres (Booker et al., 2020; Stapley et al., 2017; Stevison & McGaugh, 2020). Genomic regions of low recombination rate exhibit higher *F*_ST_ metrics, which leads to an increase in the rate of false positives (Booker et al., 2020). Introgressive hybridization or a recent history of shared ancestry between populations has also been linked to false‐positive results (Excoffier et al., 2009; Pfeifer et al., 2020). In addition, linkage disequilibrium (LD) is of relevance (Price et al., 2008) but is challenging to detect without a genome assembly. For GEA methods in particular, the results also can be impacted by the specific environmental variables chosen, their resolution and the challenges to detect smaller‐scale heterogeneity (Rellstab et al., 2015). All of these phenomena may produce significant ‘outlier’ loci in GSO or significant associations in GEA, but would not necessarily be ‘adaptive’ loci, highlighting the importance of distinguishing both concepts. Regardless of the cause of false positives in the search for adaptive loci, it is clear that limiting their impact should be a priority when identifying candidate loci. To this aim, we applied several techniques, including the combination of methods using the geometric mean of *q*‐values and simulation of spurious environmental variables. For *O. neglectus*, our analyses do not support the existence of introgression among clades (Figure S27), but bottlenecks might be present (Figure S30), further increasing the likelihood of outliers not due to selection. There is insufficient genomic information for this species to assess the impact of genome‐wide variation in mutation and recombination. Ultimately, more genomic resources are necessary to completely disentangle natural selection from the confounding factors mentioned above.

*O. neglectus* is a valuable system for exploring the influence of gene flow and environmental variability on local adaptation due to several factors: the broad distribution of the species, spanning a wide latitudinal gradient of environmental factors (O'Neill, 1985); the species' brooding development (Creese, 1986); and the differential level of gene flow mediated by the presence or absence of kelp rafting (Salloum et al., 2020). The first challenge of studying a system with such a strong background population structure is dealing with missing data when calling variants (Graham et al., 2020). For this reason, it was important to test different parameter combinations and population maps when identifying SNPs, particularly in the NZ‐wide scale. The resulting dataset for this broader scale might be more representative of conserved regions of the genome, as we aimed to reduce missing data among clades with the applied filters. By also analysing the North Island, South Island and southern populations separately, determining SNPs within each group (as opposed to simply splitting the original SNP dataset), we were able to reduce missing data, and identified a larger number of loci that are variable within clades, and not only between them (Figures S17–S23, Table S2). These within‐clade datasets are presumably more representative of the full diversity of variable loci within each clade and hence were used at these scales in our genome scans.

### 4.1 Genome scans for outliers

PCAdapt detected a larger number of ‘outlier’ markers than Bayescan in all spatial scales, although both methods detected an unusually high number of loci NZ‐wide. Combining these methods using the geometric mean of *q*‐values returned intermediate values in the three smaller scales, but an even larger number of ‘outlier’ markers NZ‐wide. This larger number of combined ‘outlier’ markers indicates that all these loci had relatively high support in at least one of the methods, leading the geometric mean to be below the threshold (0.05). Overall, the two methods did not detect a large proportion of loci in common (intersection, Figure 2), although we did not necessarily expect the same loci to be detected by such different methods given the different assumptions of the underlying models. The combined ‘outlier’ markers exhibit lower differentiation among nearby populations at all scales than the patterns exhibited by all the other loci (Figure 2c–e, Figures S17–S23). This could be interpreted within the adaptive hypothesis to indicate that similar selective forces may act on nearby populations and make them more genetically similar than expected based on the patterns from all the other loci. These findings are different from those of another system with strong background population structure, the black tiger shrimp *Penaeus monodon*, in which loci consistently detected across four genome scan and genotype–environment association tests displayed the same pattern of population structure as neutral loci (Vu et al., 2020).

### 4.2 Genotype–environment association

Genotype–environment association methods applied to the NZ‐wide context either identified a large proportion of GEA loci (BayeScEnv method), or no associations at all (RDA method) (Table 3), but the lack of high‐confidence GEA loci NZ‐wide suggests low support for GEA loci identified with BayeScEnv. In the environmentally narrow NI and SI contexts, few significant loci were identified, none of them high confidence. In the southern scale, where population structure is weakest, but where broad environmental variation still exists, both methods identified significant GEA loci, and 86 of these are high confidence (Table 3), most of which are associated with latitudinally varying factors. The relatively low number of high‐confidence GEA loci may be a consequence of the reduced environmental variation when compared to the NZ‐wide scale.

### 4.3 Controlling false positives

There has previously been much consideration of the possible ways to control false‐positive rates in genome scans (Lotterhos et al., 2017). Our novel strategy proposes to first use geometric means to combine *q*‐values across methods and then, for the GEA methods, to remove loci that are also associated with 5% or more of the random variables. This approach was useful to find candidate loci that are less likely to be false positives and have spurious associations, although we acknowledge that true‐positive loci are likely to have been excluded in the process. Previous work has shown that considering the intersection of genome scan methods tends to focus the result on a true signal (de Villemereuil et al., 2014, Figure S12). Here, the intersection of all four genome scan methods results in 58 candidate loci for the southern scale.

### 4.4 Implications of findings for *O. neglectus*

Our analyses of population structure and relative migration support the subdivision of *O. neglectus* into three, strongly isolated clades. There is higher connectivity within the South clade due to gene flow driven by kelp rafting, and minimal connectivity between clades. Populations in the South clade have a greater proportion of low‐frequency alleles compared to populations in the North and Central clades. This may be consistent with a bottleneck in the North and Central clades, as previously indicated by a mitochondrial DNA marker (Salloum et al., 2020). Here, the allele frequency spectra‐based stairway plots support a recent bottleneck, although we note that allele frequency spectra patterns can be driven by a variety of factors, including demographic changes, population structure and selection (Gattepaille et al., 2013; Keinan & Clark, 2012; Ronen et al., 2013).

In agreement with our initial hypothesis, we saw evidence for local adaptation in *O. neglectus*. Initially, our GSO and GEA analyses appeared to identify a very large proportion of candidate loci potentially linked to local adaptation in the NZ‐wide and in the southern scale. However, this perspective was modified with more careful and conservative consideration of these candidate loci, by combining *q*‐values and removing all but the high‐confidence GEA results using a novel approach (Table 3). By focusing attention on these high‐confidence GEA loci, it became apparent that these could not be strongly supported at the NZ‐wide scale. However, and in contrast to our initial expectation that higher rates of migration might lead to lower evidence of local adaptation, there remained considerable evidence for potential local adaptation at the southern scale. An examination of the allele frequencies of these loci (Figure S33) may help to explain this phenomenon, as it is apparent that most GEA loci at the broad scale derive from changes in fixation of alternate alleles, and they do not systematically vary in frequency across the environmental gradient from north to south. At the point of fixation, it is impossible to distinguish if drift or local adaptation were responsible for fixing such loci. In contrast, at the southern scale, most associations reflect gradual changes in allele frequency, which provide a more favourable context for association with the environment. Ultimately, the strongest evidence from this study reveals that higher gene flow among southern populations (driven by kelp rafting) does not appear to have removed the opportunity for local adaptation, and has in fact made it easier to detect candidate loci potentially under selection.

Most of the environmental variables tested are correlated with latitude, or to factors in the marine environment that vary latitudinally. Interestingly, migration rate and geographical distance did not show strong correlation, not supporting isolation‐by‐distance and providing stronger evidence for the influence of the environment in driving some of the differentiation observed in these loci (Figure S29). There was a match of one *O. neglectus* high‐confidence GEA locus to the *A. granulata* transcriptome, but unfortunately it was to a non‐annotated region. *A. granulata* belongs to the same family as *O. neglectus*, but is not closely related, thus loci that match its transcriptome are likely to be conserved genes. As for many non‐model species, *O. neglectus* currently has insufficient genomic resources to enable identification of the function of candidate loci. As more genomes are annotated and genomic resources become increasingly accessible, it might soon be possible to recover the exact function of candidate loci even in non‐model species. Such resources are paramount for a more thorough understanding of the genetic basis of local adaptation in natural populations (Hoban et al., 2016), helping characterize species' responses to environmental variability. Ultimately, this is required for efficient management of biodiversity in the upheaval of our changing climate.

## 5 CONCLUSIONS

The use of genomic scans for outliers and genotype–environment association methods has been providing great insight into understanding the distribution of potentially adaptive variation in natural populations, which is crucial for appropriately managing biodiversity in a changing climate (Flanagan et al., 2018). These methods appear to generally perform well when assessing simple population scenarios, but much development is still needed to attain the same standards for less ‘ideal’, non‐model populations (Benestan et al., 2016; Booker et al., 2020; Forester et al., 2016). In *O. neglectus*, a novel method was used to increase the probability of identifying a reliable set of candidate loci for selection, but also indicates that it is still a challenge to correctly identify true positives in this confounding scenario of population structure. However, our methods have provided powerful insight within the southern region, showing that the potential for local adaptation has not been eliminated by gene flow. Furthermore, among the high‐confidence GEA loci found, we identified a functional genomic region that could have an adaptive role in the evolution of these populations. More empirical assessments and comparisons of ‘challenging’ populations can help with outlining the variation expected under real scenarios, prompting further development to better accommodate such diversity of the natural world. In addition to the ongoing growth in the availability of genomic resources, advances in methodological approaches will enable more comprehensive understanding of local adaptation and its underlying causes in the wild, leading to a better understanding of the complex responses of organisms to changes in their environment.

## CONFLICT OF INTEREST

The authors have no conflict of interest to declare.

## AUTHORS' CONTRIBUTIONS

All authors helped plan the research and contributed to writing the manuscript; P.d.V. also helped with statistical analysis and designed the novel statistical approach used in this work; P.M.S. undertook sampling, laboratory work, statistical analyses and writing. All authors gave final approval for publication.

## Supporting information

Data S1

## Data Availability

Data and scripts used for the analyses are available from the figshare https://doi.org/10.17608/k6.auckland.c.5906135.v1 (Salloum et al., 2022).
