# Supplementary material for: Finding the adaptive needles in a population‐structured haystack: A case study in a New Zealand mollusc
Source: J Anim Ecol. 2022 Mar 30;91(6):1209–21. doi: 10.1111/1365-2656.13692 (PMC9311215; doi:10.1111/1365-2656.13692)
Supplement: Supplementary file 1 — Data S1 [file JANE-91-1209-s001.pdf]

Supplementary Material

This material is organised as Supporting Information (containing more details on the genotyping-by-sequencing and SNP calling methods), Supporting Figures (containing additional figures to support results) and Supporting Tables (containing additional tables with details on datasets and results).

Supporting Information 1

Sampling, DNA extraction, and Genotyping-by-Sequencing

DNA extraction and initial checks of concentration and purity were undertaken following the protocol described in Salloum *et al*. (2020). DNA concentration was normalised to 20-100ng/ul, after which samples were submitted to AgResearch Invermay, Mosgiel, New Zealand, for genotyping-by-sequencing (GBS). The enzymes Pst-I and Msp-I were used for the GBS. Fragments were size selected in the range of 193-318 with Pippin Prep (SAGE Science, Beverly, Massachusetts, USA) and 188 samples were single-end sequenced (1x101bp) on an Illumina HiSeq2500 utilizing v4 chemistry and two lanes of an Illumina Hi-Seq flow cell (169 single samples and 19 replicates within and between lanes, comprising a randomly chosen replicate per population within the same lane and three randomly chosen replicates across lanes). For quality checks, two controls per lane were also included. The resulting single-end reads were tested for quality by AgResearch, using a custom pipeline (available at https://github.com/AgResearch/DECONVQC), and a FastQC v0.10.1 report (Andrew 2010). Adapters were removed with cutadapt (Martin 2011). The resulting Fastq files were provided to us, one for each lane.

De-multiplexing, parameter optimisation and SNP calling

We de-multiplexed fragments and called SNPs *de-novo* using Stacks v.2.4 (Catchen et al. 2011; Catchen et al. 2013). To de-multiplex, the Stacks module "process radtags" was used, with inline_null barcodes and the following settings: -c (clean data and remove any read with an uncalled base); -q (discard reads with low quality scores); -r (rescue barcodes and Rad-tags); and -t 92 (trim fragments to 92 base pairs). None of the 188 barcoded samples failed. To assemble rad-tags and call SNPs, parameters for the "ustacks" and "cstacks" modules were initially explored using a smaller dataset with sixteen samples. We defined the population map in the Stacks "populations" module by grouping these samples according to their region of collection (four samples from the North Island, two from the Central New Zealand, five from the South Island, and five from the Sub-Antarctic Islands). A variety of rad-tag assembly and SNP-calling parameters were tested, following recommendations from Rochette & Catchen (2017): the number of reads required to start a new putative allele was kept constant at three (m = 3), while the number of mismatches allowed between two alleles of a heterozygote (M) was always kept equal to the number of mismatches between any two alleles of the population (n), but tested with values ranging from one to nine in different runs (M=n=1 to M=n=9, in nine different runs) (Fig. S1A). In addition, considering prior knowledge regarding similarity within populations and differences between populations (Salloum et al. 2020), we performed a run with m=3, M=2, n=4, aiming at keeping differences within populations realistic based on previous analyses (up to four mismatches between any two alleles of the population, but a maximum of two mismatches between heterozygote alleles).

To detect as many SNPs as possible while limiting erroneous calls, we compared the total number of SNPs, rad-tags (*i.e.*, assembled sequences, each 92 bases long) and sites (*i.e.*, total number of bases assembled into contigs) obtained for each different setup (Fig. S1A), and then tested the three most promising parameter combinations on the complete dataset

(188 samples), with a population map defining the 16 populations. The influence of the r parameter in the Populations module (percentage of individuals in a population required to retain a locus in the population) was checked by running r = 0.5 and r = 0.8. Runs were performed with and without a minor allele frequency filter set to 0.05. Parameters for subsequent analyses were selected as the combination that minimised the number of rad-tags with large numbers of SNPs while retaining a high number of rad-tags with one or two SNPs in order to minimise SNPs that represented collapsed repeat regions (Fig. S1B). The SNPs in the NZ-wide dataset were called with m = 3, M = 2 and n = 4 and using a population map that split populations by clades (North, Central and South), forcing rad-tags to be present in at least 50% of the samples within each clade (settings in populations module: p = 3, r = 0.5) and using a minor allele frequency filter of 0.05. This ensured a more 'conserved' dataset, in which missing data among clades was much reduced (Fig. S3A-B). The SNPs in the southern, NI and SI dataset were called with m = 3, M = 2, n = 4, p = 1, r = 0.8, minor allele frequency filter of 0.05, and a population map defining each population (ten populations in the southern dataset and four in each of the NI and SI datasets, Fig. S3C-E). To avoid strong linkage disequilibrium in all datasets, one random SNP per rad-tag was selected, using this option of the Populations module in Stacks. Pairwise squared Euclidean genetic distances of individuals and technical replicates were calculated using the R package Adegenet v. 2.1.1 (Jombart 2008; Jombart & Ahmed 2011) (Fig. S2).

Technical replicates were then removed, by keeping only the replicated sample with the larger number of SNPs. All datasets were further filtered by removing individuals with more than 90% missing data (two individuals from the Christchurch population) with the SNP_GBS filter pipeline (Alexander 2018), and removing rad-tags with more than 30% missing data with VCFtools v. 0.1.14 (Danecek et al. 2011). This resulted in removal of one population (Christchurch) from all but the NZ-wide dataset.

References

Alexander, A. (2018) GBS_SNP_filter v1.x.x. *Github repository*. Available online at: https://github.com/laninsky/GBS_SNP_filter

Andrew, S. (2010) FastQC: a quality control tool for high throughput sequence data. Available online at: http://www.bioinformatics.babraham.ac.uk/projects/fastqc/

Catchen, J., Hohenlohe, P.A., Bassham, S., Amores, A. & Cresko, W.A. (2013) Stacks: an analysis tool set for population genomics. *Molecular Ecology*, **22,** 3124-3140. 10.1111/mec.12354

Catchen, J.M., Amores, A., Hohenlohe, P., Cresko, W. & Postlethwait, J.H. (2011) Stacks: building and genotyping Loci de novo from short-read sequences. *G3: Genes | Genomes | Genetics*, **1,** 171-182. 10.1534/g3.111.000240

Danecek, P., Auton, A., Abecasis, G., Albers, C.A., Banks, E., DePristo, M.A., Handsaker, R.E., Lunter, G., Marth, G.T., Sherry, S.T., McVean, G., Durbin, R. & Genomes Project Analysis Group (2011) The variant call format and VCFtools. *Bioinformatics,* **27,** 2156-2158. 10.1093/bioinformatics/btr330

Jombart, T. (2008) adegenet: a R package for the multivariate analysis of genetic markers. *Bioinformatics*, **24,** 1403-1405. 10.1093/bioinformatics/btn129

Jombart, T. & Ahmed, I. (2011) adegenet 1.3-1: new tools for the analysis of genome-wide SNP data. *Bioinformatics*, **27,** 3070-3071. 10.1093/bioinformatics/btr521

Martin, M. (2011) Cutadapt removes adapter sequences from high-throughput sequencing reads. *EMBnet journal,* **17,** 10-12. https://doi.org/10.14806/ej.17.1.200

Rochette, N.C. & Catchen, J.M. (2017) Deriving genotypes from RAD-seq short-read data using Stacks. *Nature Protocols,* **12,** 2640-2659. 10.1038/nprot.2017.123

Salloum, P.M., De Villemereuil, P., Santure, A.W., Waters, J.M. & Lavery, S.D. (2020) Hitchhiking consequences for genetic and morphological patterns: the influence of

kelp-rafting on a brooding chiton. *Biological Journal of the Linnean Society,* **130,** 756-777. 10.1093/biolinnean/blaa073/5859172

Supporting Figures

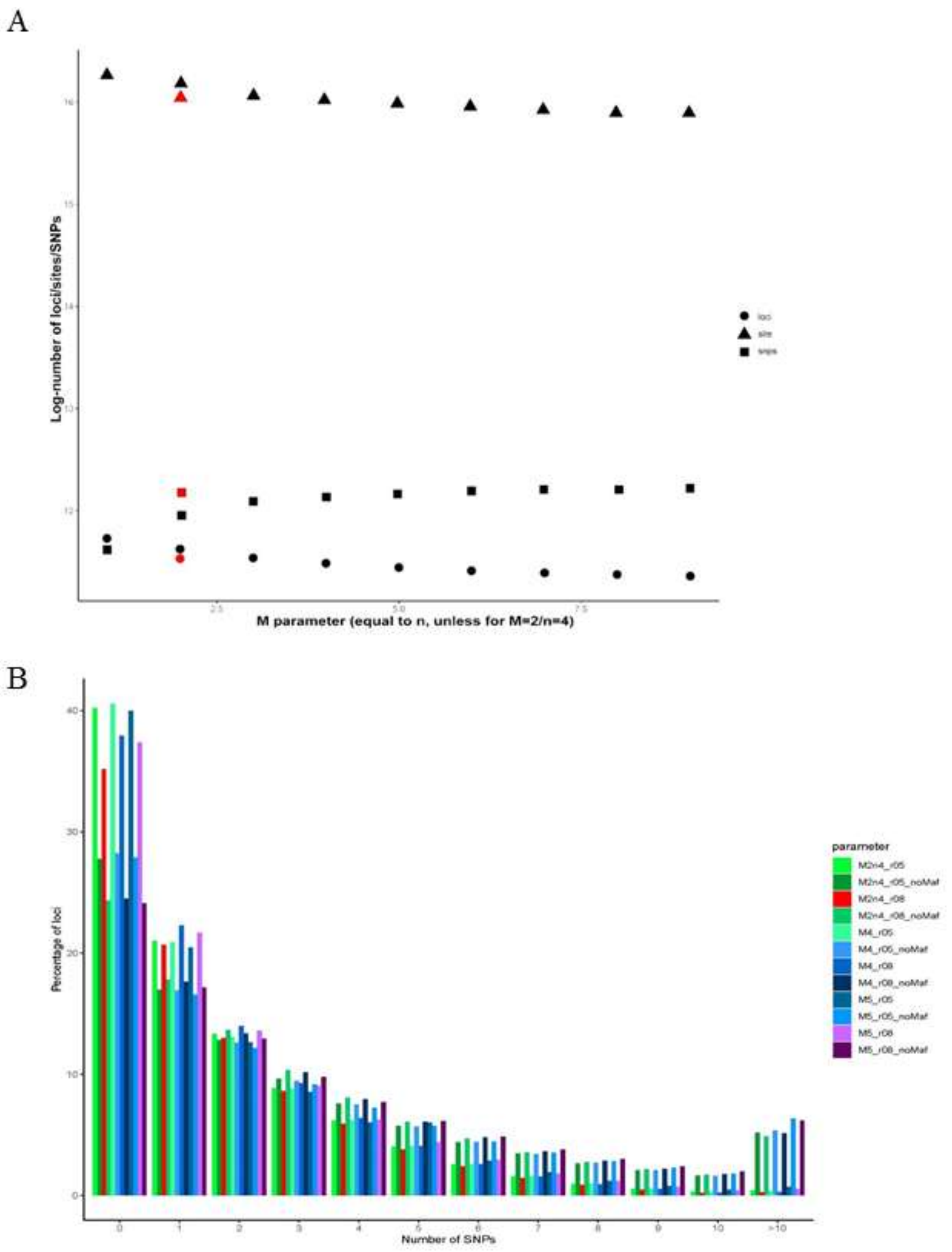

Fig. S1 Parameter optimization for *de-novo* SNP calling. A) Number of sites, loci (rad-tags) and SNPs for different parameter changes in the Stacks software using a trial dataset of sixteen individuals, grouped by geographic region (four in the North Island, two in the

Central New Zealand, five in the South Island and five in the Subantarctic Islands). The y axis has the log transformation of the number of loci, i.e., rad-tags (circles), sites (triangles), and SNPs (squares), and the x axis has the parameter changes for M and n (in red are the results for the parameters of choice, M = 2 and n = 4); B) Number of SNPs per locus for different parameter changes in Stacks, and with the addition of the minor allele frequency (maf) filter, using all samples. The y axis has the percentage of loci with 0 to more than 10 SNPs, and the x axis has the number of SNPs (from 0 to more than 10). The results for the parameters of choice are indicated in red (M = 2, N = 4, r = 0.8, maf = 0.05), which yielded more loci with fewer SNPs and fewer loci with a large number of SNPs. For the relatively few loci with more than one SNP, one SNP per rad-tag was randomly selected, to exclude SNPs that would be in very strong linkage disequilibrium.

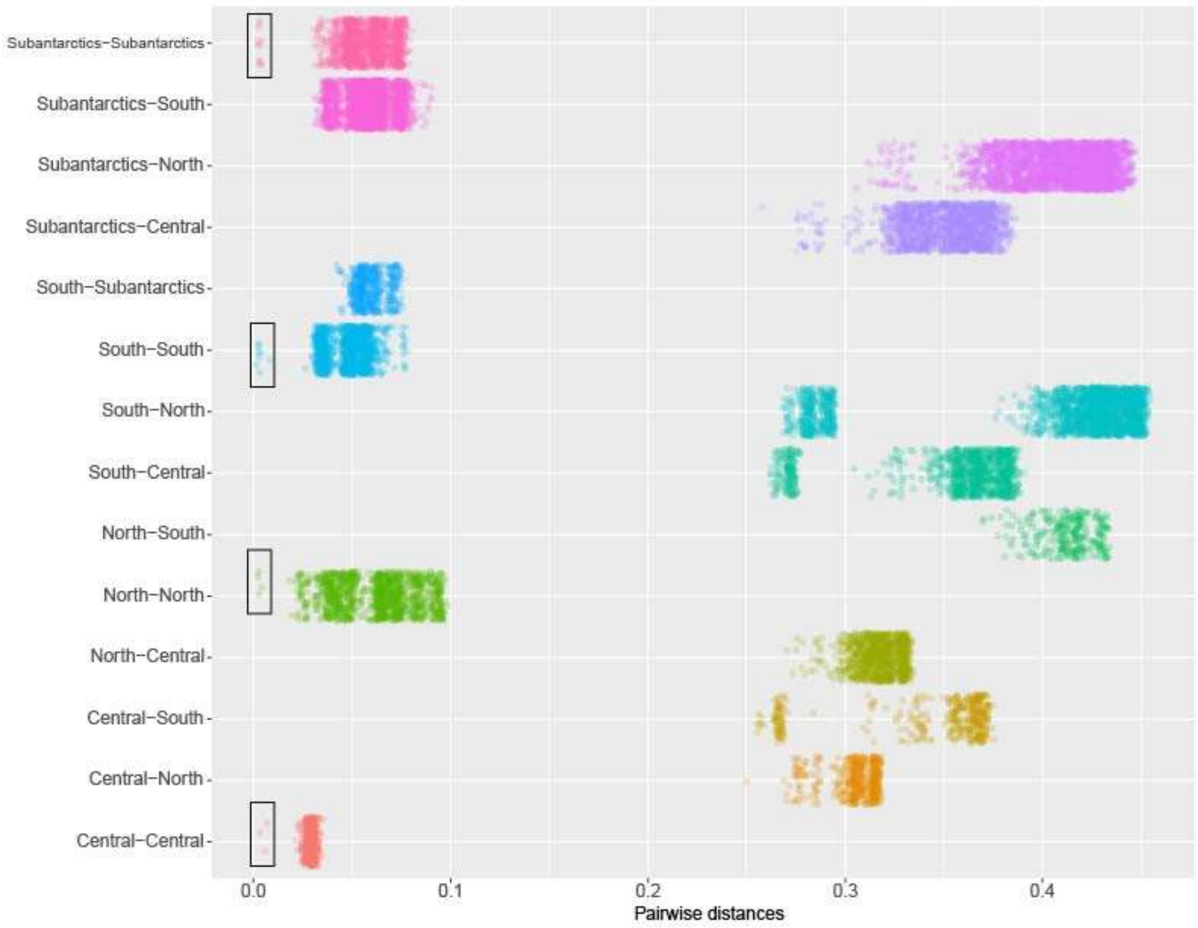

Fig. S2 Individual pairwise squared Euclidian genetic distances of all individuals, including technical replicates, calculated using the R package Adegenet v. 2.1.1 (Jombart, 2008; Jombart and Ahmed, 2011). Black rectangles indicate pairwise comparisons between technical replicates. Technical replicates were removed from the main analyses (with all samples), by keeping only the replicated sample with the larger number of SNPs from the parameter optimization steps. Samples are grouped by geographic region. ‘Subantarctics’ includes the Sub-Antarctic Auckland Islands (AU), Campbell Islands (CA), Antipodes Islands (AN), Bounty Islands (BO), and Chatham Islands (CH). ‘South Island’ includes Christchurch (CR), Dunedin (DU), Akatore (AK), Curio Bay (CU), and Stewart Island (ST). ‘Central’ includes Wellington (WE) and Cape Palliser (CP); ‘North’ includes Russell (RU), Auckland (TI), Coromandel (NC), and East Cape (EC).

A. NZ-wide dataset – three-clades population map

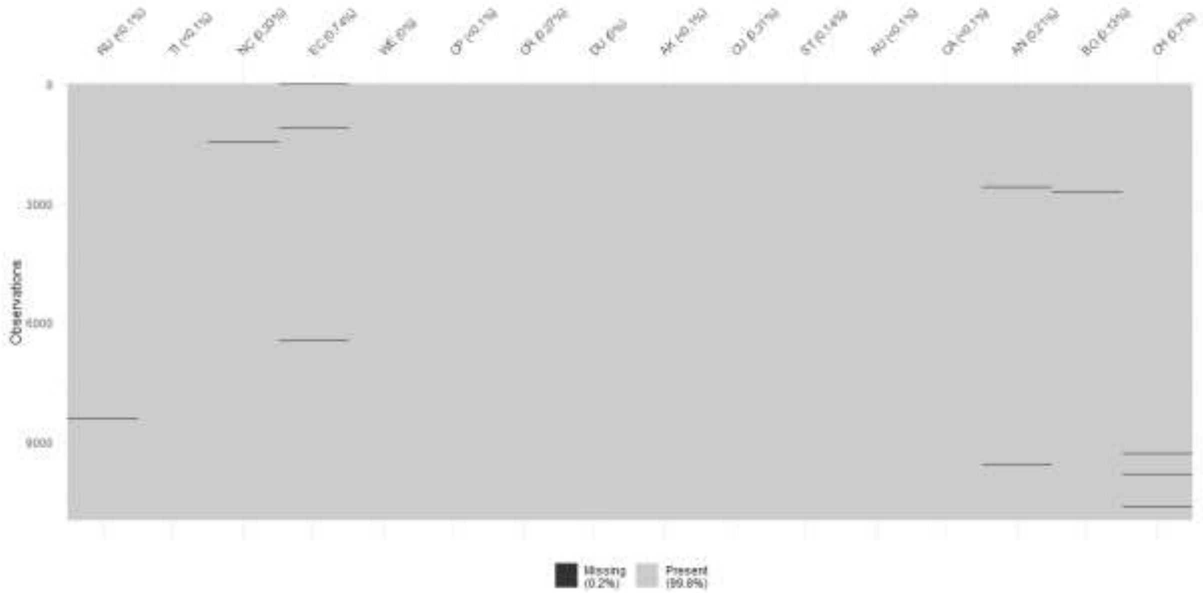

B. NZ-wide dataset – 16-populations population map

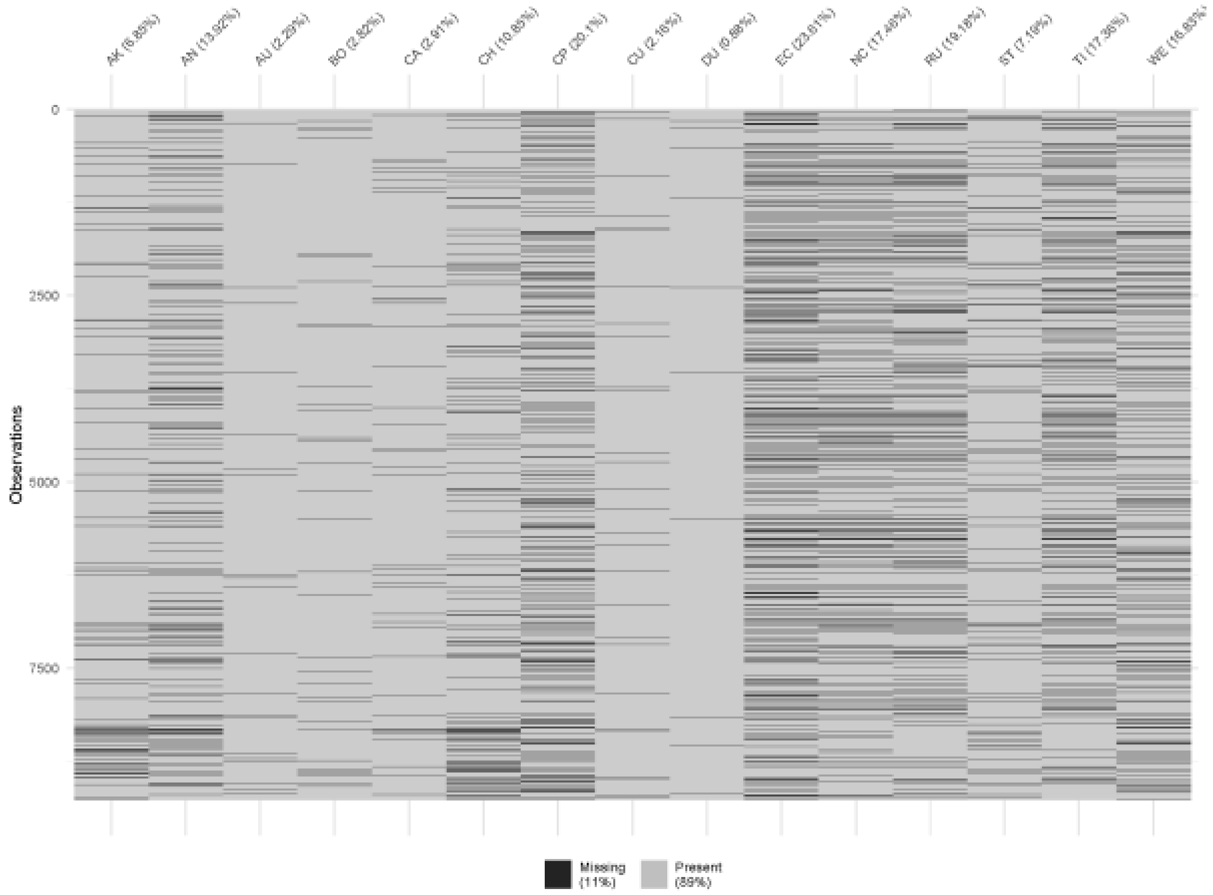

C. NI-specific dataset

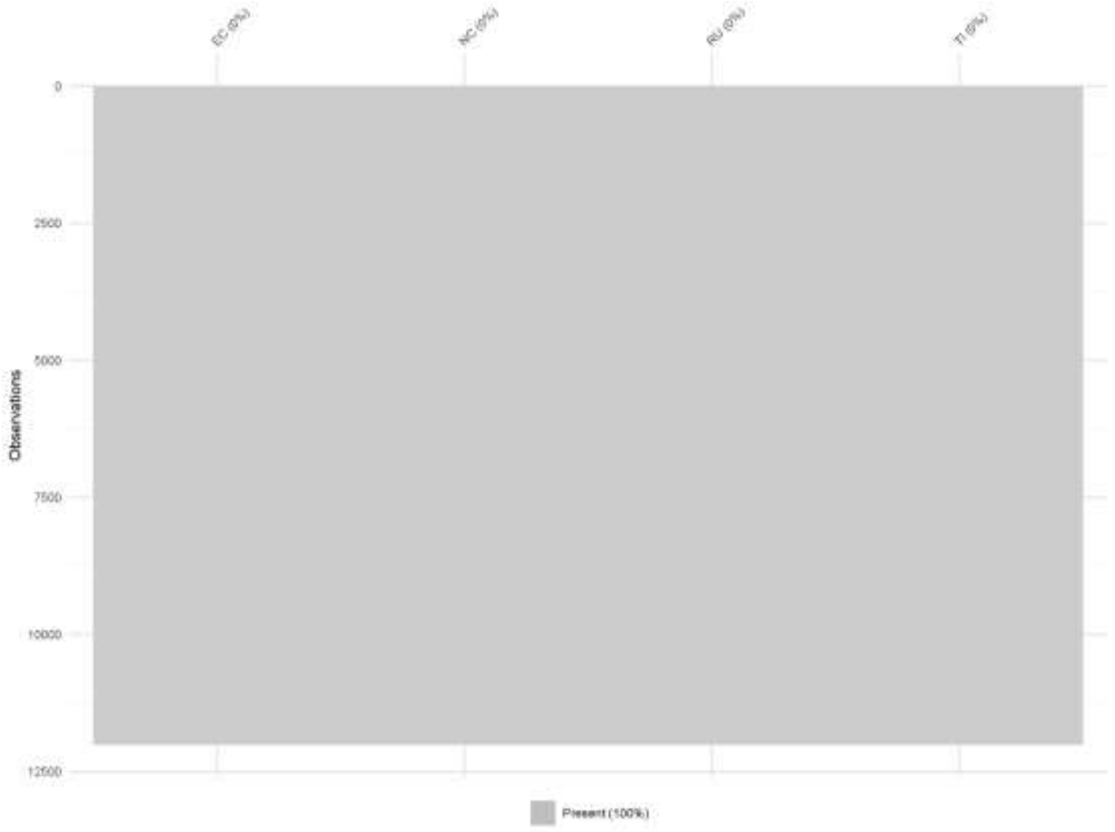

D. SI-specific dataset

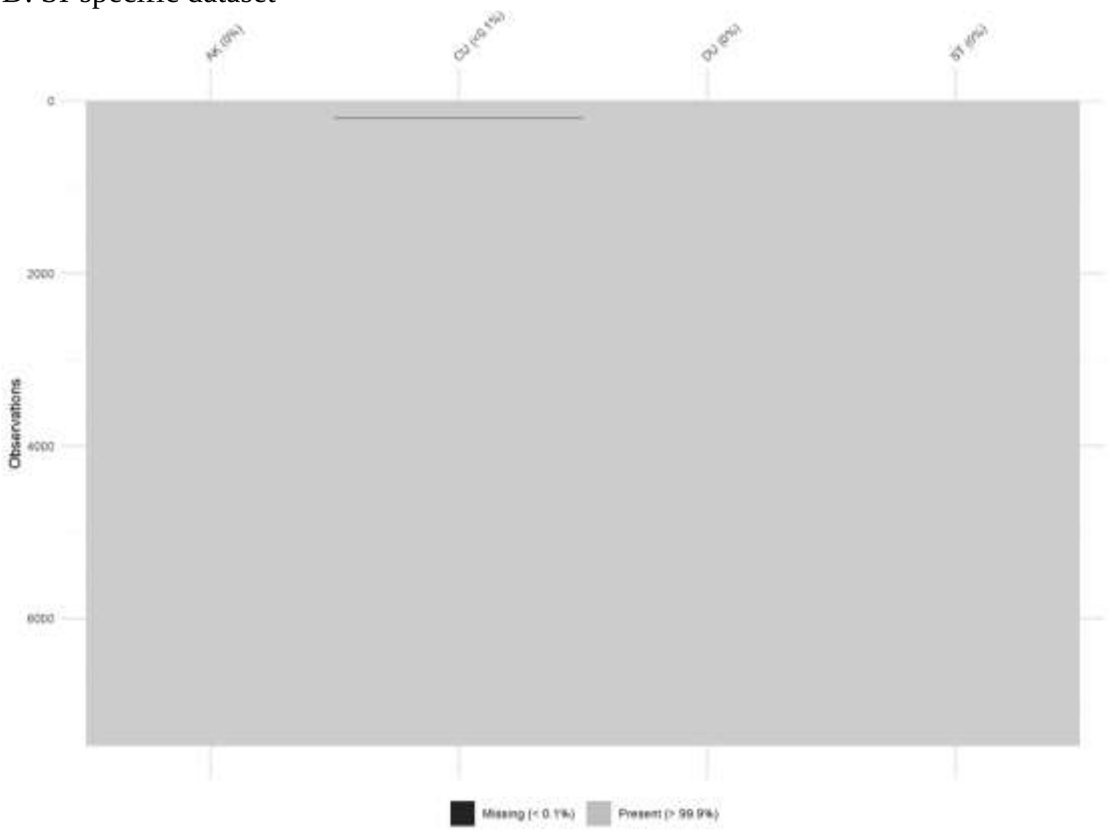

E. southern dataset

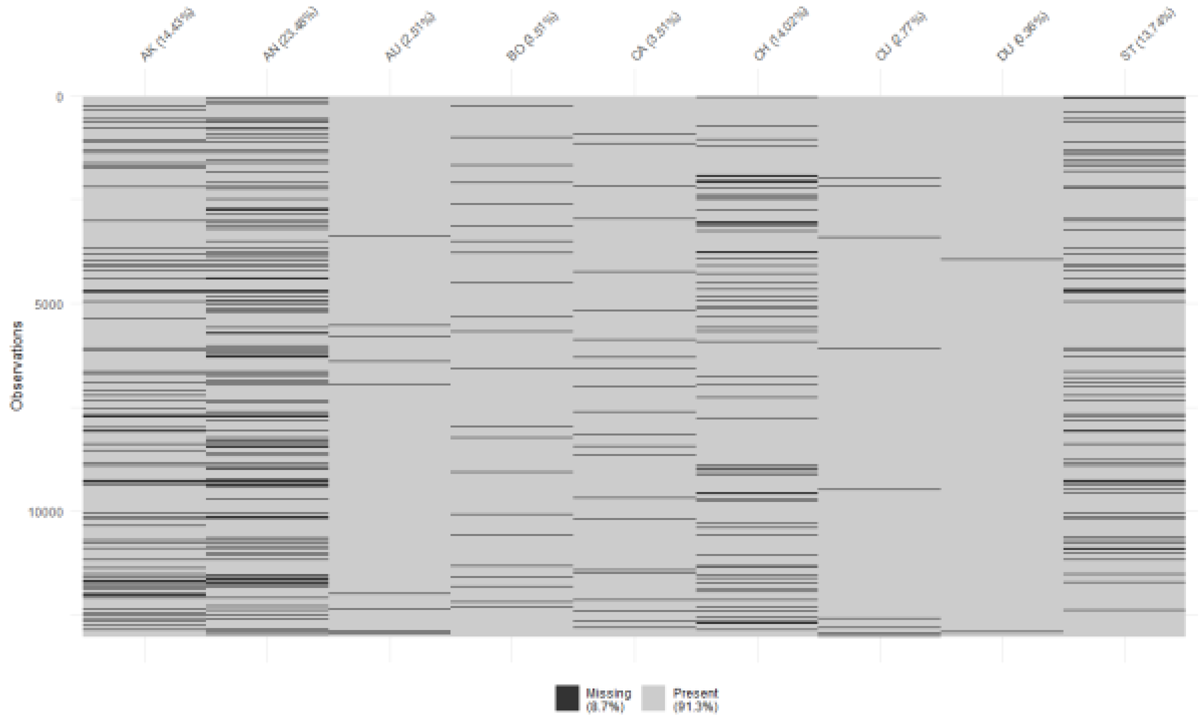

Fig. S3 Percentage of loci missing per population. Missing data is shown as dark grey bands. A) NZ-wide datasets with 10,987 SNPs (0.2% missing), called with a population map identifying the three clades (and not the 16 populations). B) NZ-wide dataset with 9,275 SNPs (11% missing), called with a population map identifying each of the 16 populations (not used in analyses, given the lower rates of missing data in A). C) NI dataset with 12,012 SNPs (0% missing), called with a population map identifying four North Island populations; D) SI dataset with 7,476 SNPs (0.1% missing), called with a population map identifying four South Island populations; southern dataset with 13,004 SNPs (8.7% missing), called with a population map identifying the 10 southern populations (9 populations are shown due to the exclusion of Christchurch during filtering).

A. NZ-wide dataset

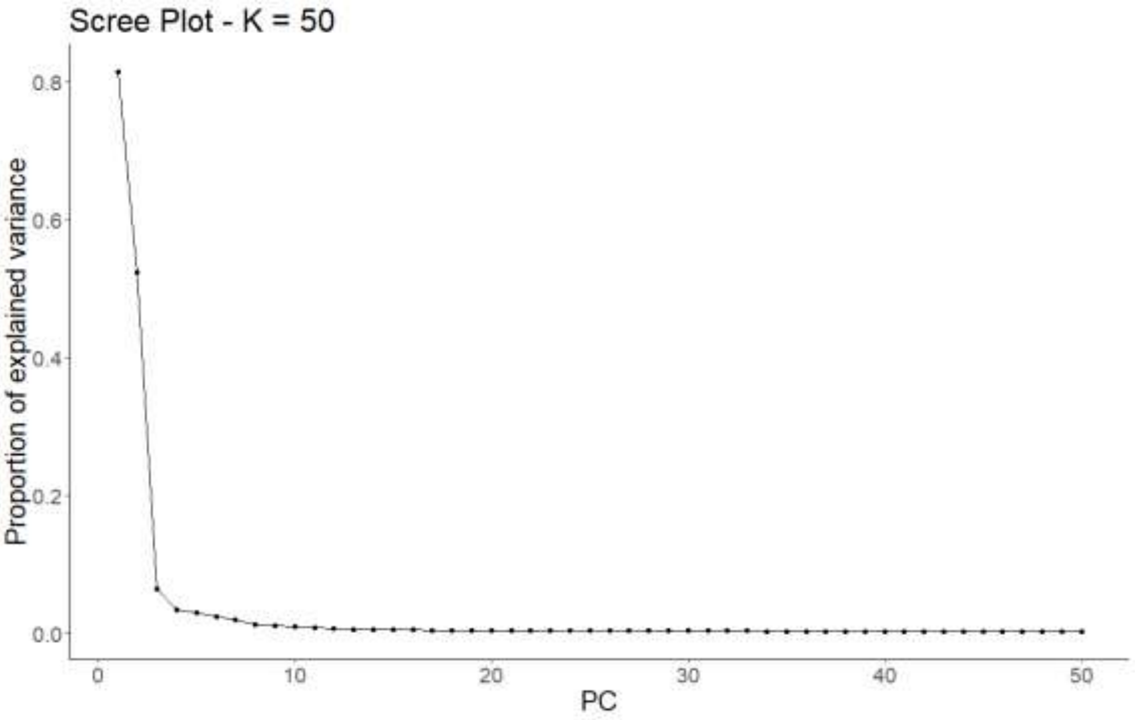

B. NI-specific dataset

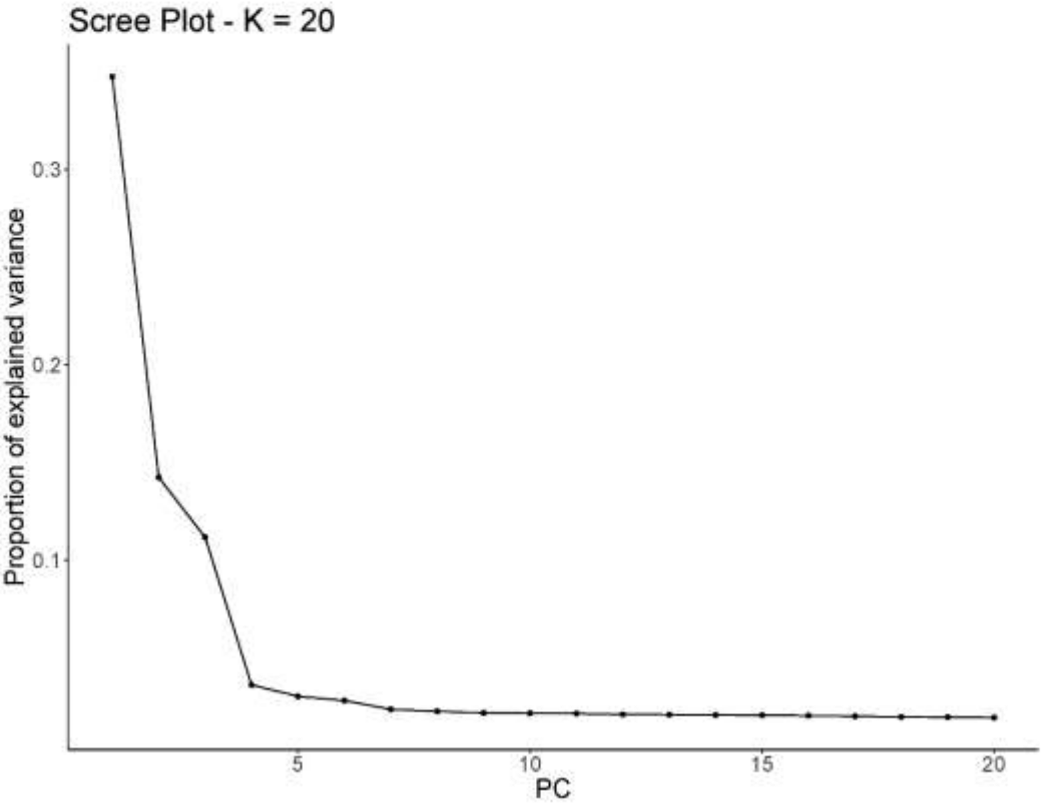

C. SI-specific dataset

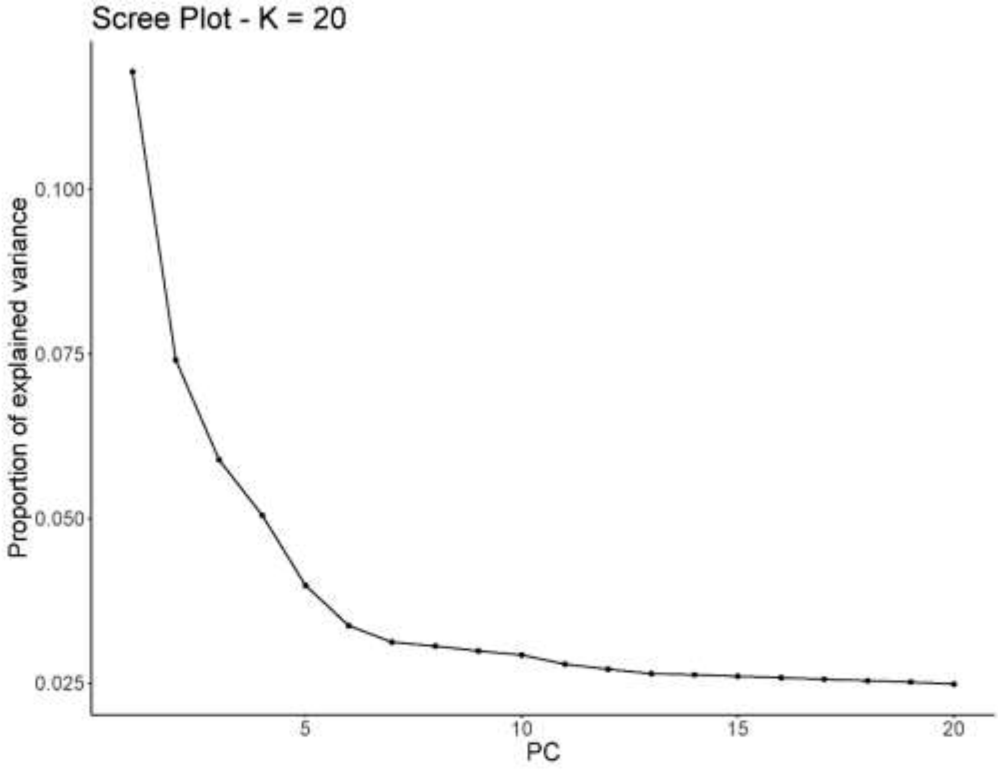

D. southern dataset

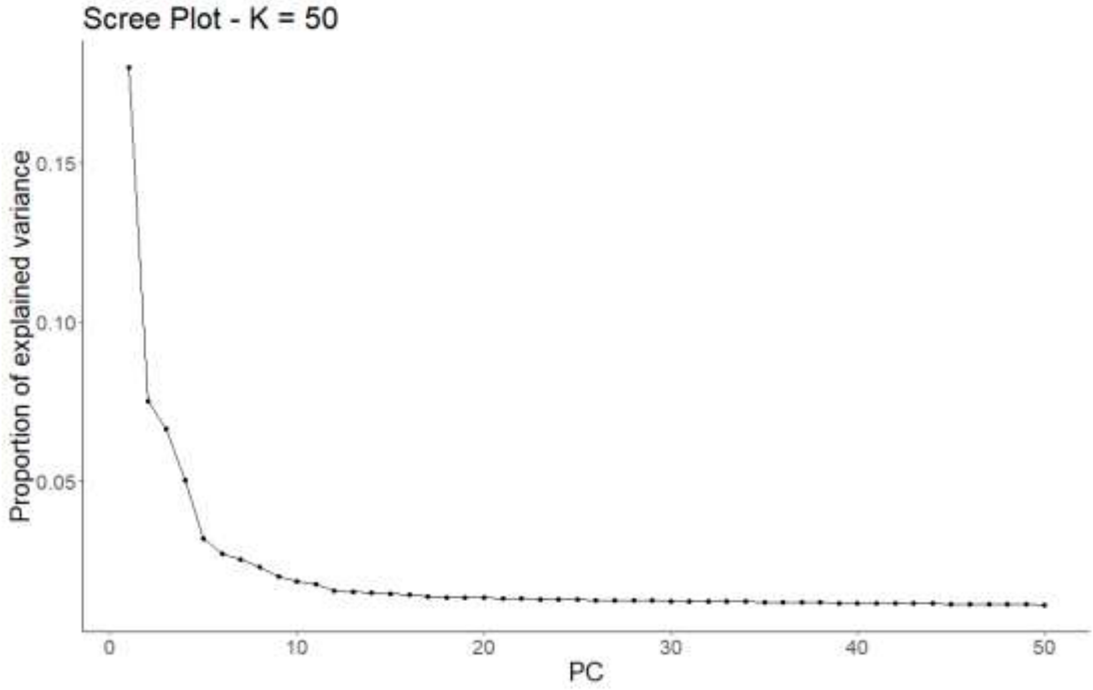

Fig. S4 Scree plot of PCAdapt, showing proportion of variance explained by each principal component (PC), in a run with PC = 50 or PC = 20. A) NZ-wide; B) NI; C) SI; D) southern.

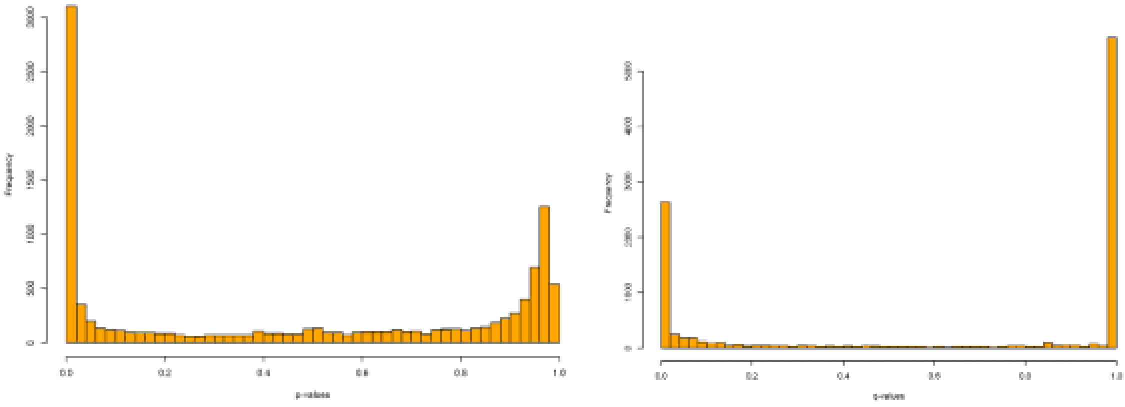

Fig. S5 Distribution of *p*-values (left) and *q*-values (right) for the PCAdapt analysis on the NZ-wide dataset.

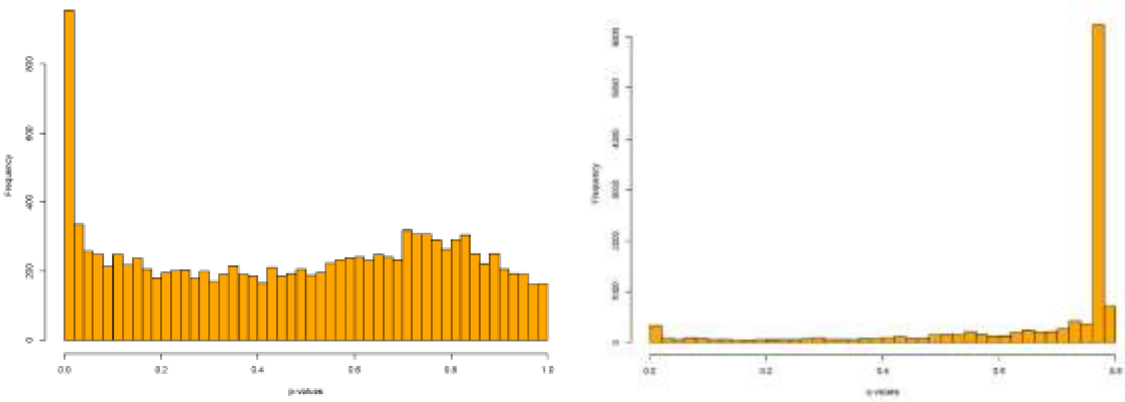

Fig. S6 Distribution of *p*-values (left) and *q*-values (right) for the PCAdapt analysis on the NI_specific dataset.

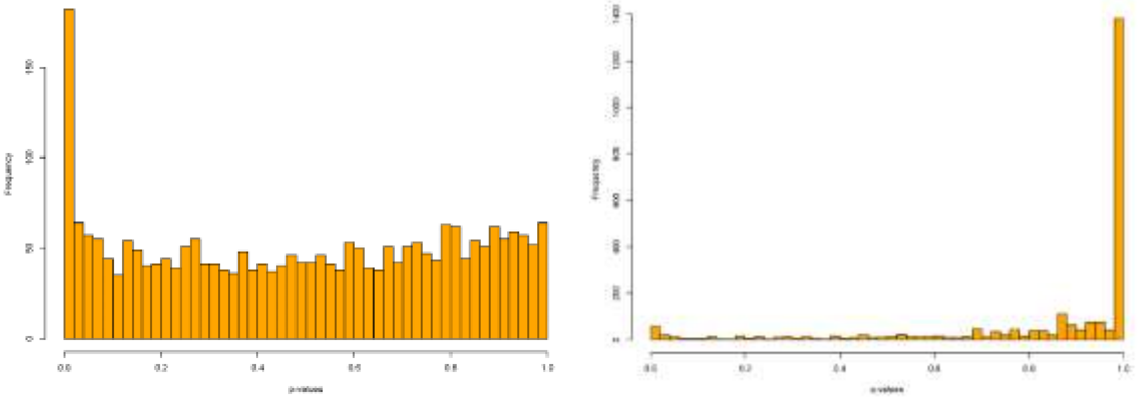

Fig. S7 Distribution of *p*-values (left) and *q*-values (right) for the PCAdapt analysis on the NI_split dataset.

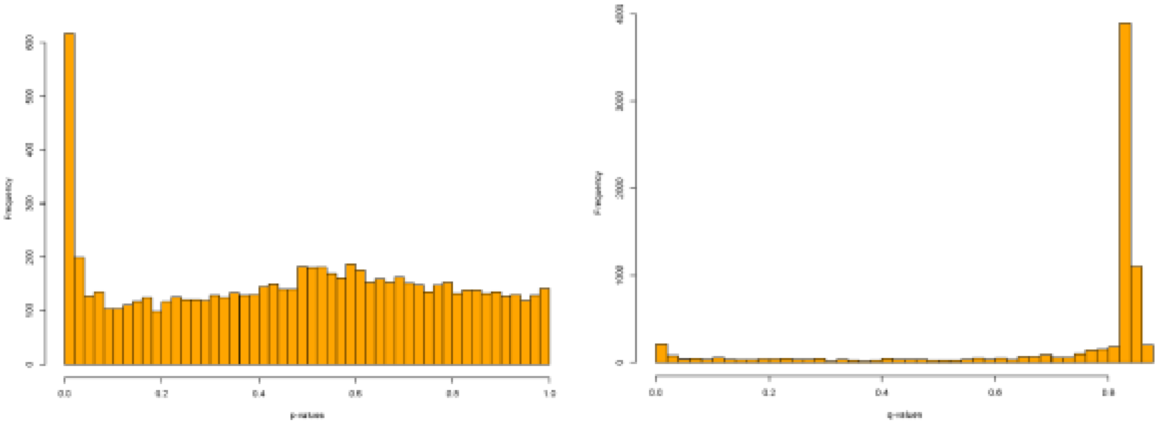

Fig. S8 Distribution of *p*-values (left) and *q*-values (right) for the PCAdapt analysis on the SI_specific dataset.

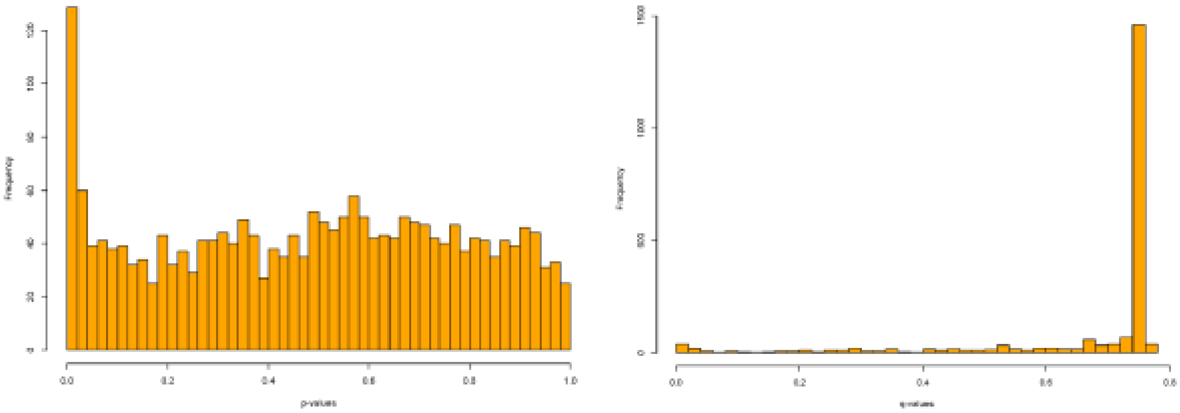

Fig. S9 Distribution of *p*-values (left) and *q*-values (right) for the PCAdapt analysis on the SI_split dataset.

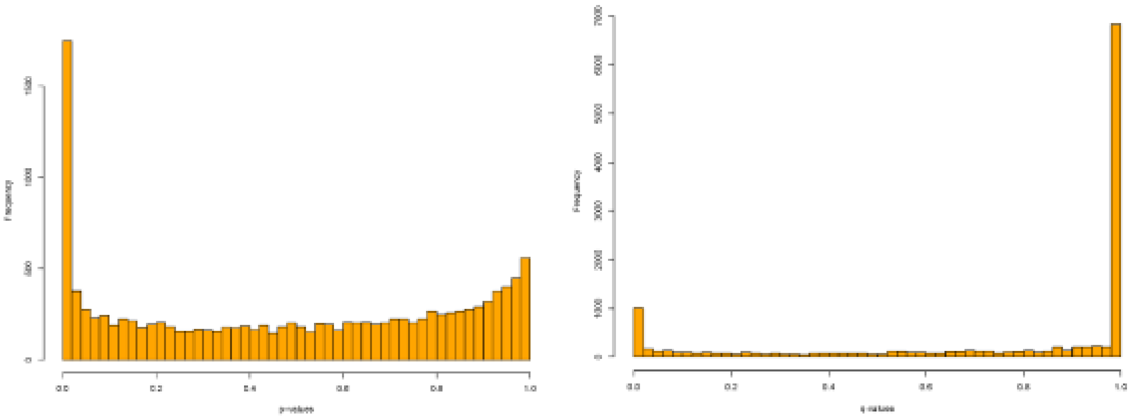

Fig. S10 Distribution of *p*-values (left) and *q*-values (right) for the PCAdapt analysis on the Southern_specific dataset.

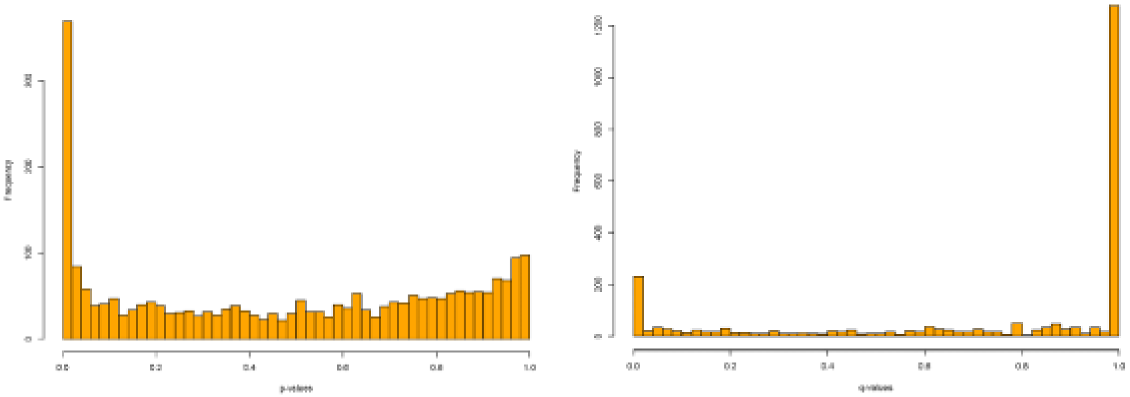

Fig. S11 Distribution of *p*-values (left) and *q*-values (right) for the PCAdapt analysis on the Southern_split dataset.

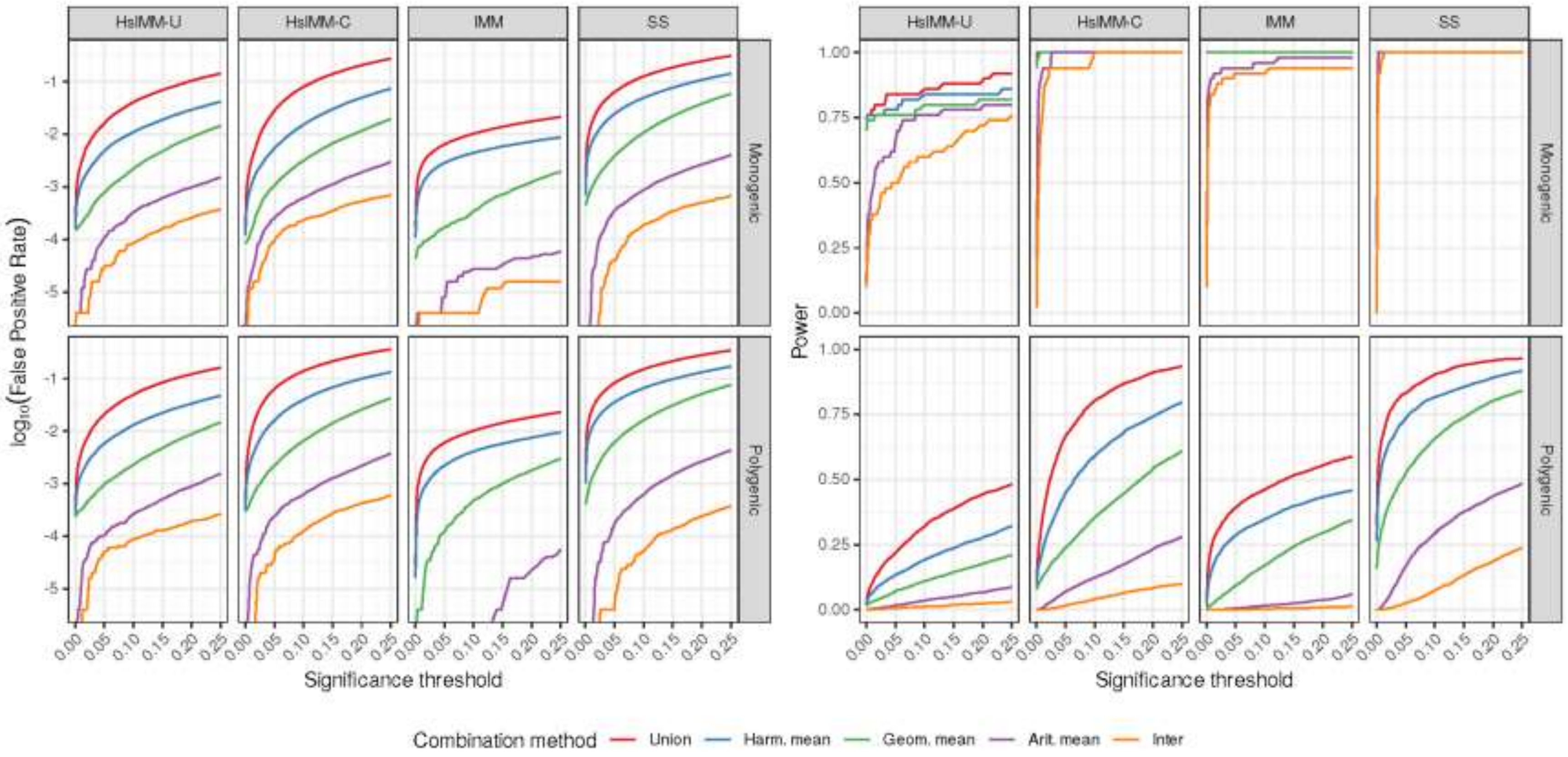

Fig. S12 Using data from de Villemereuil et al. (2014). Simulations showing false positive rates (left) and power (right) following different ways of considering genome scan results across methods (union: any method is significant; intersection: all methods are significant; Harm., Geom. and Arit. Mean: respectively taking harmonic, geometric and arithmetic mean, then assessing significance), and over different demographic contexts (HsIMM-U:and HsIMM-C: highly structured isolation with migration model, with selection correlated with demographic history, -U, or over an

environmental gradient, -C; IMM: isolation with migration model; SS: stepping-stone model). See Table 1 in de Villemereuil et al. (2014) for further details on models.

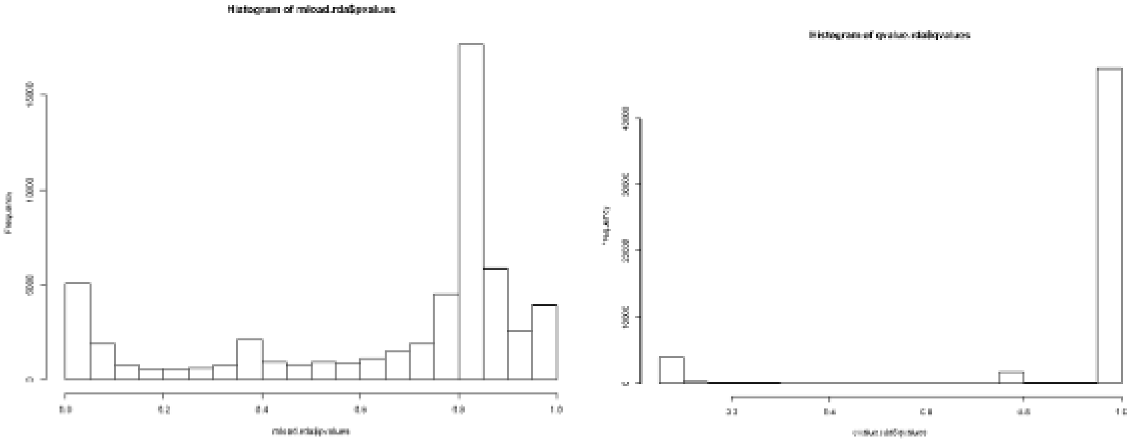

Fig. S13 Distribution of *p*-values (left) and *q*-values (right) for the RDA analysis on the NZ-wide dataset.

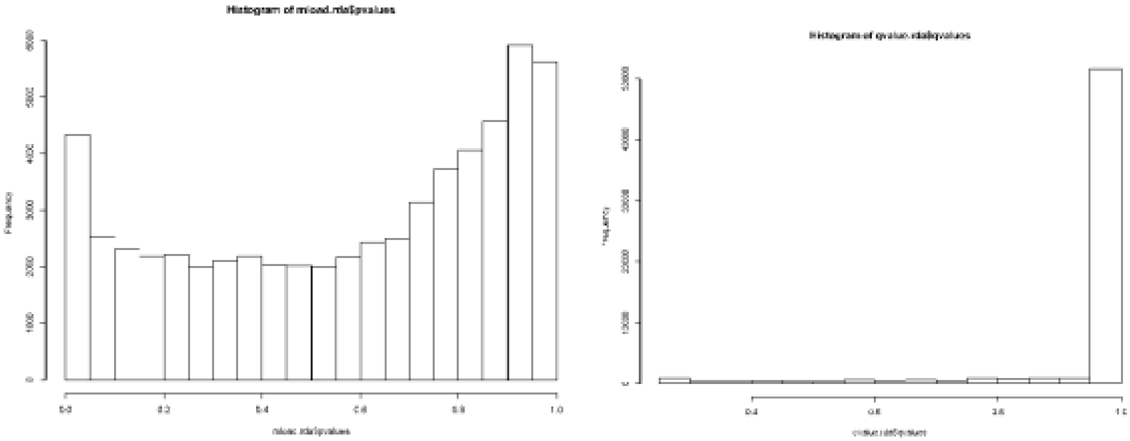

Fig. S14 Distribution of *p*-values (left) and *q*-values (right) for the RDA analysis on the NI_specific dataset.

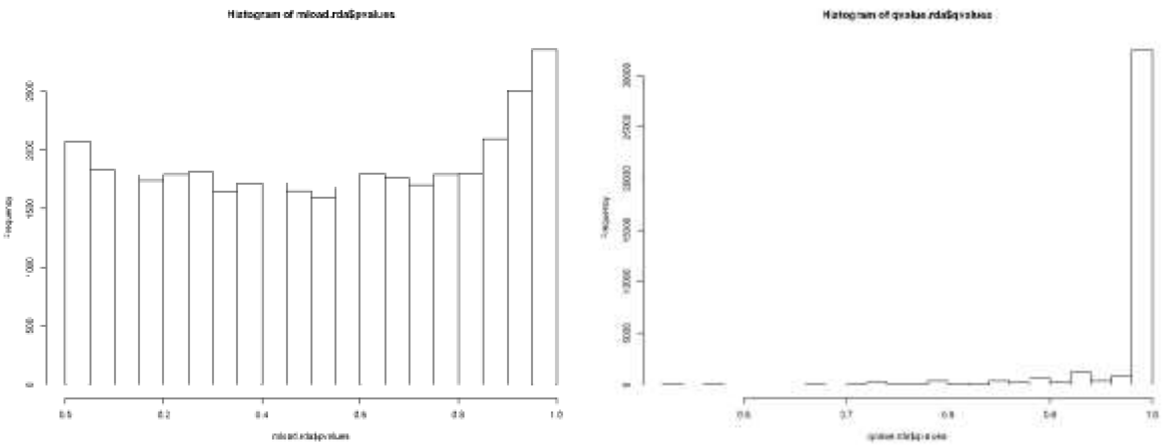

Fig. S15 Distribution of *p*-values (left) and *q*-values (right) for the RDA analysis on the SI_specific dataset.

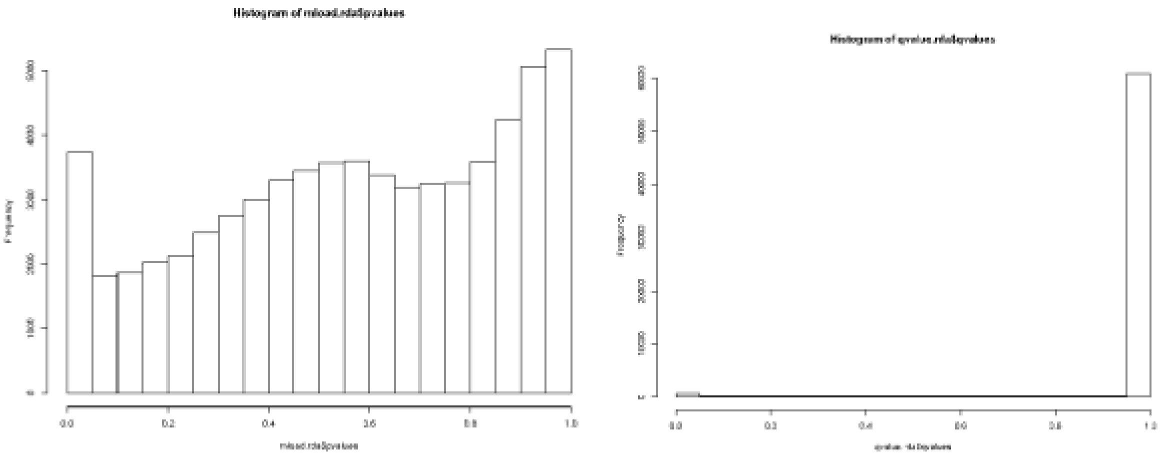

Fig. S16 Distribution of *p*-values (left) and *q*-values (right) for the RDA analysis on the Southern_specific dataset.

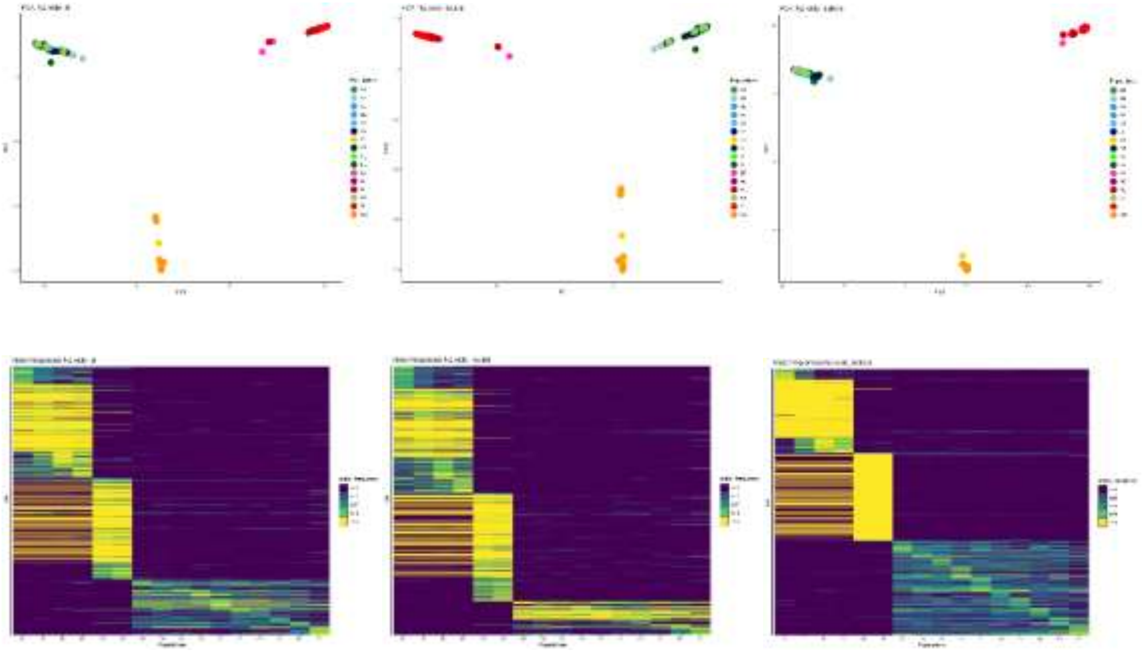

Fig. S17 NZ-wide PCA (top row) and allele frequencies (bottom row) for all loci, remaining loci after removing combined ‘outlier’ markers, and combined ‘outlier’ markers (Bayescan and PCAdapt results combined by the geometric mean of q-values, cut-off = 0.05).

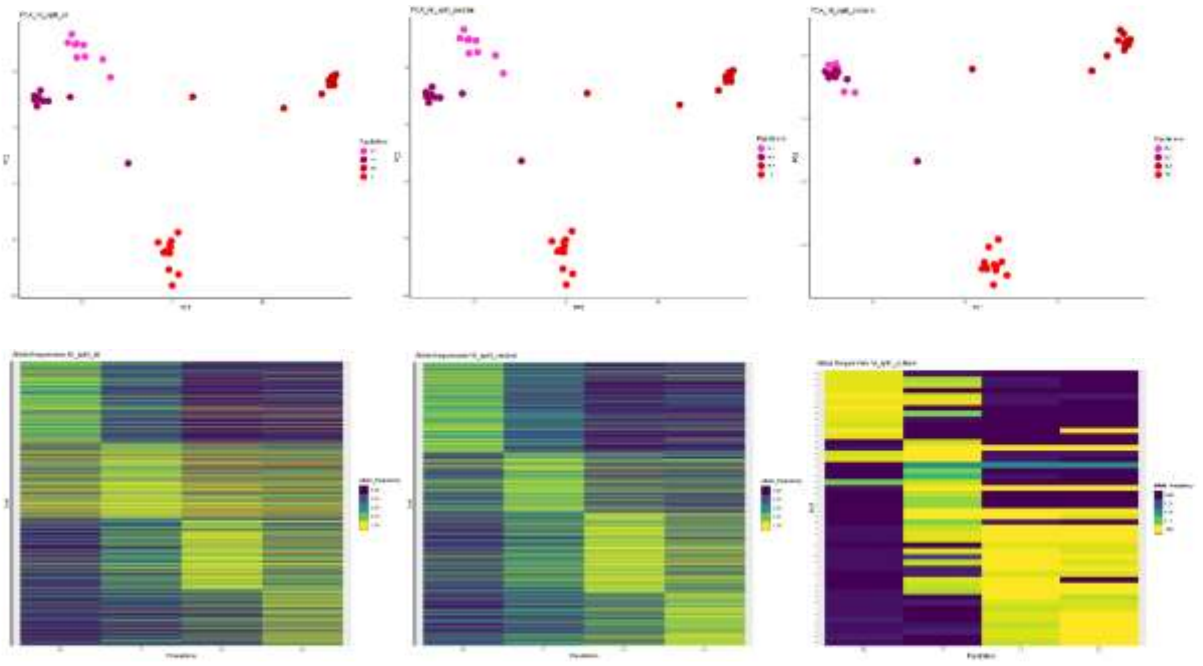

Fig. S18 North Island Split PCA (top row) and allele frequencies (bottom row) for all loci, remaining loci after removing combined ‘outlier’ markers, and combined ‘outlier’ markers (Bayescan and PCAdapt results combined by the geometric mean of q-values, cut-off = 0.05).

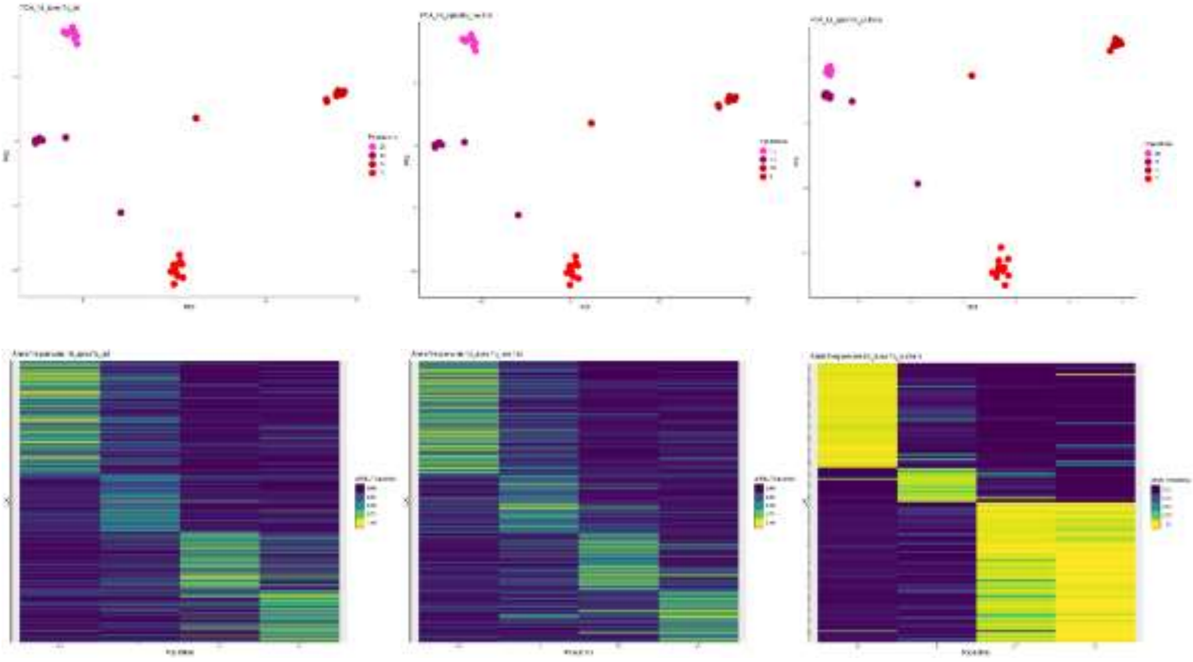

Fig. S19 North Island Specific PCA (top row) and allele frequencies (bottom row) for all loci, remaining loci after removing combined ‘outlier’ markers, and combined ‘outlier’ markers (Bayescan and PCAdapt results combined by the geometric mean of *q*-values, cut-off = 0.05).

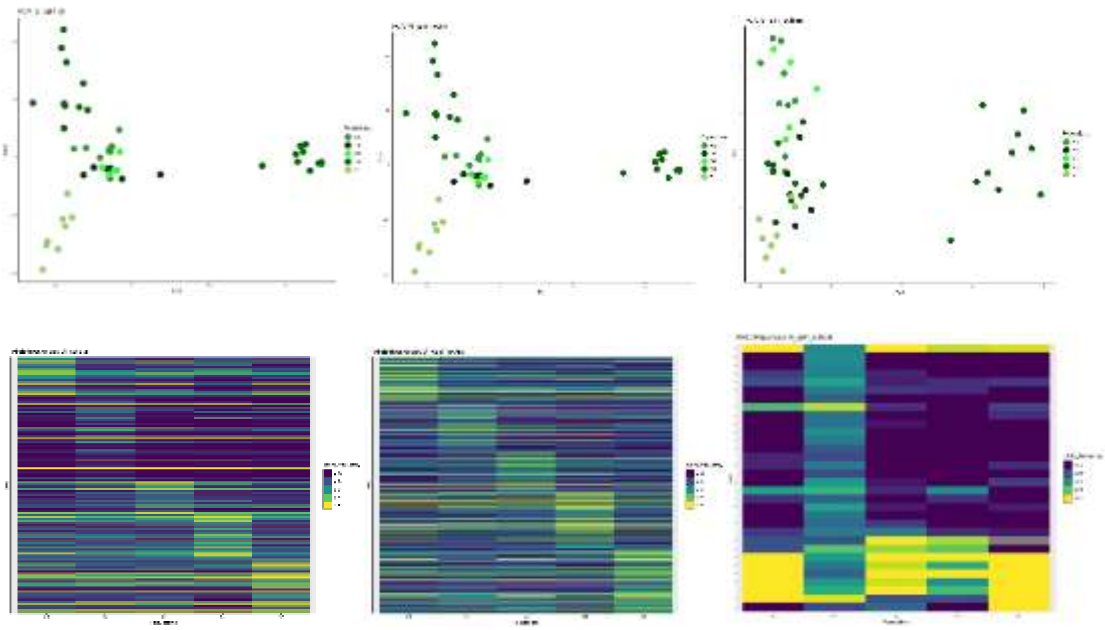

Fig. S20 South Island Split PCA (top row) and allele frequencies (bottom row) for all loci, remaining loci after removing combined ‘outlier’ markers, and combined ‘outlier’ markers (Bayescan and PCAdapt results combined by the geometric mean of *q*-values, cut-off = 0.05).

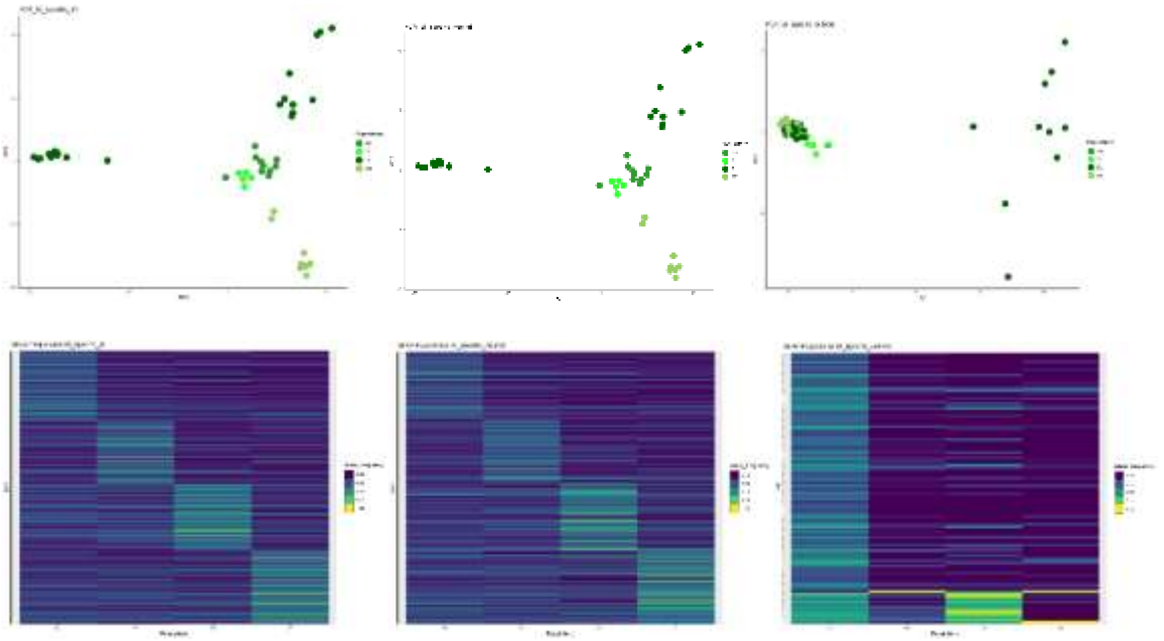

Fig. S21 South Island Specific PCA (top row) and allele frequencies (bottom row) for all loci, remaining loci after removing combined ‘outlier’ markers, and combined ‘outlier’

markers (Bayescan and PCAdapt results combined by the geometric mean of *q*-values, cut-off = 0.05).

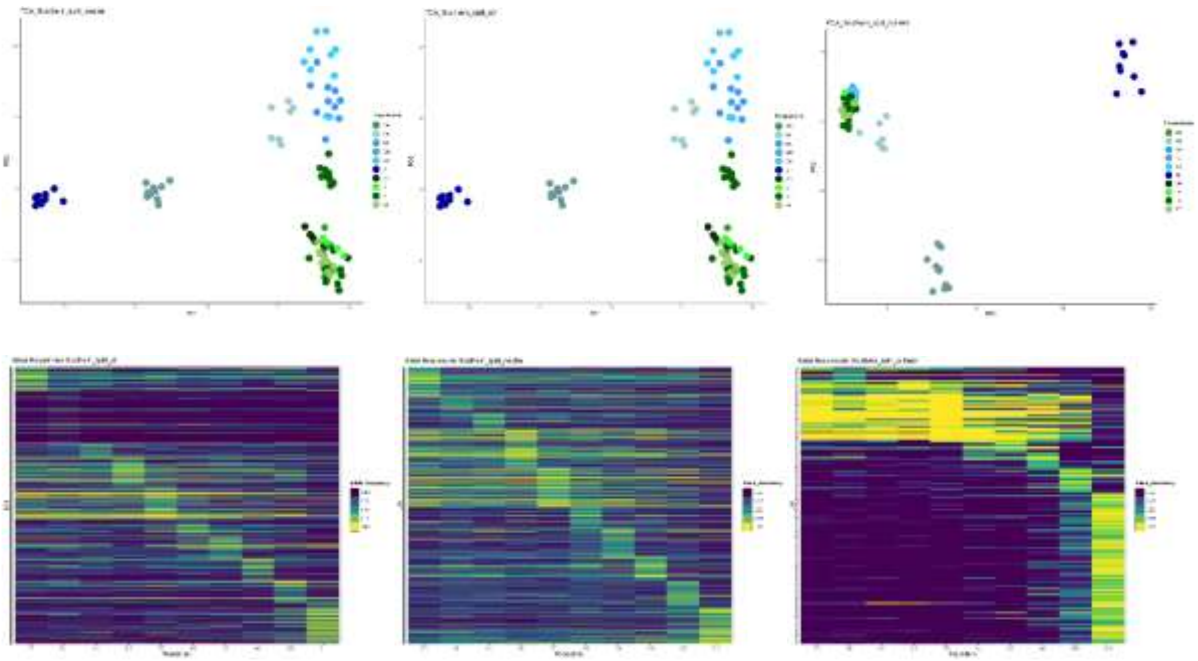

Fig. S22 Southern Split PCA (top row) and allele frequencies (bottom row) for all loci, remaining loci after removing combined ‘outlier’ markers, and combined ‘outlier’ markers (Bayescan and PCAdapt results combined by the geometric mean of *q*-values, cut-off = 0.05).

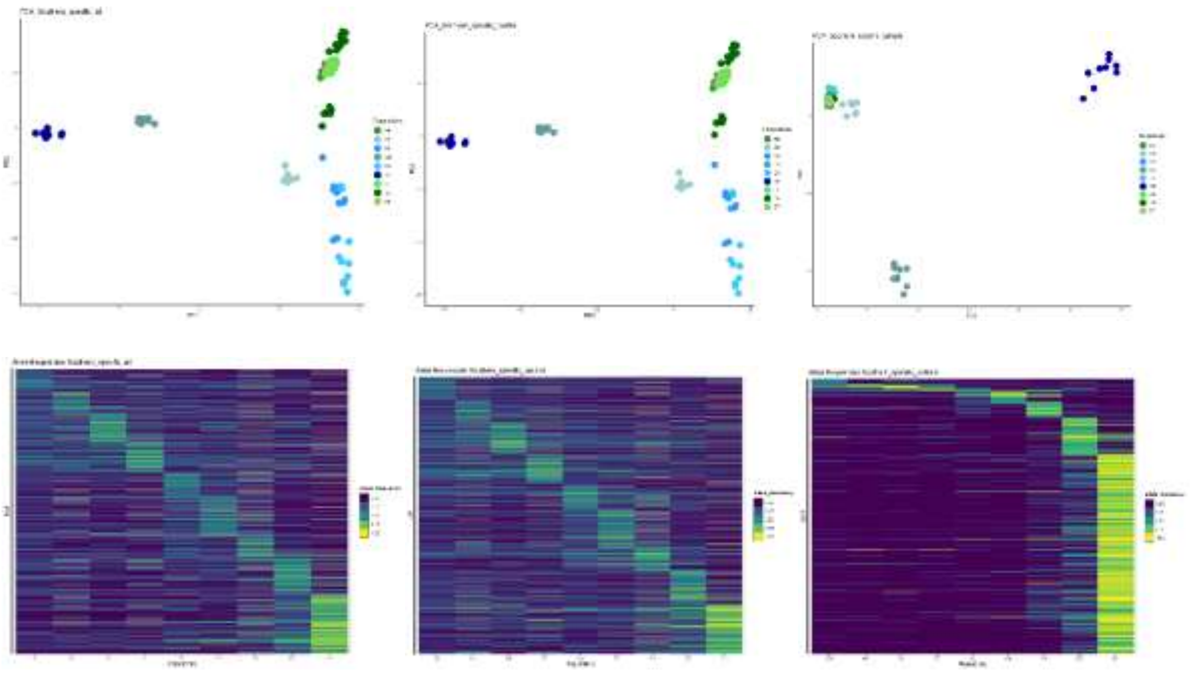

Fig. S23 Southern Specific PCA (top row) and allele frequencies (bottom row) for all loci, remaining loci after removing combined ‘outlier’ markers, and combined ‘outlier’ markers (Bayescan and PCAdapt results combined by the geometric mean of *q*-values, cut-off = 0.05).

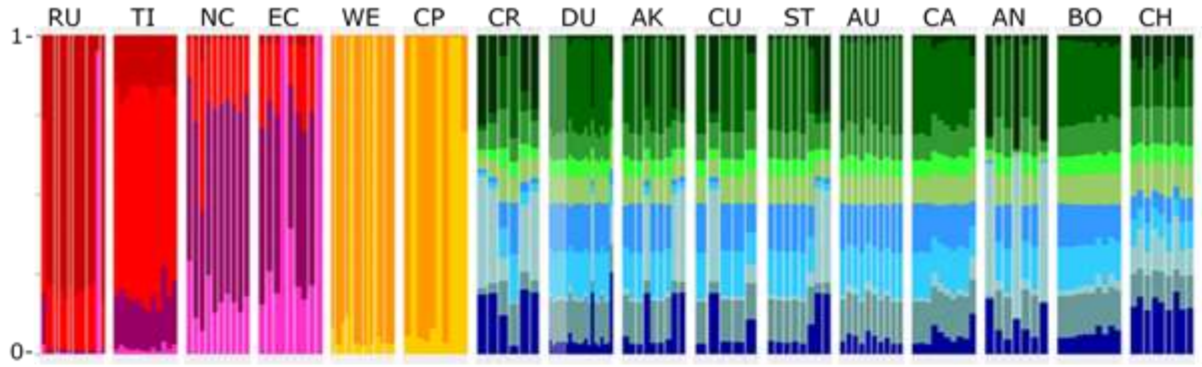

Fig. S24 Population assignment plot NZ-wide using all 10,987 loci (k = 16). Colour scheme follows the map in Fig 1. Population key: RU = Russell, TI = Auckland, NC = Coromandel, EC = East Cape, WE = Wellington, CP = Cape Palliser, CR = Christchurch, DU = Dunedin, AK = Akatore, CU = Curio Bay, ST = Stewart Island, AU = Auckland Islands, CA = Campbell Island, AN = Antipodes Islands, BO = Bounty Islands, CH = Chatham Islands.

A B

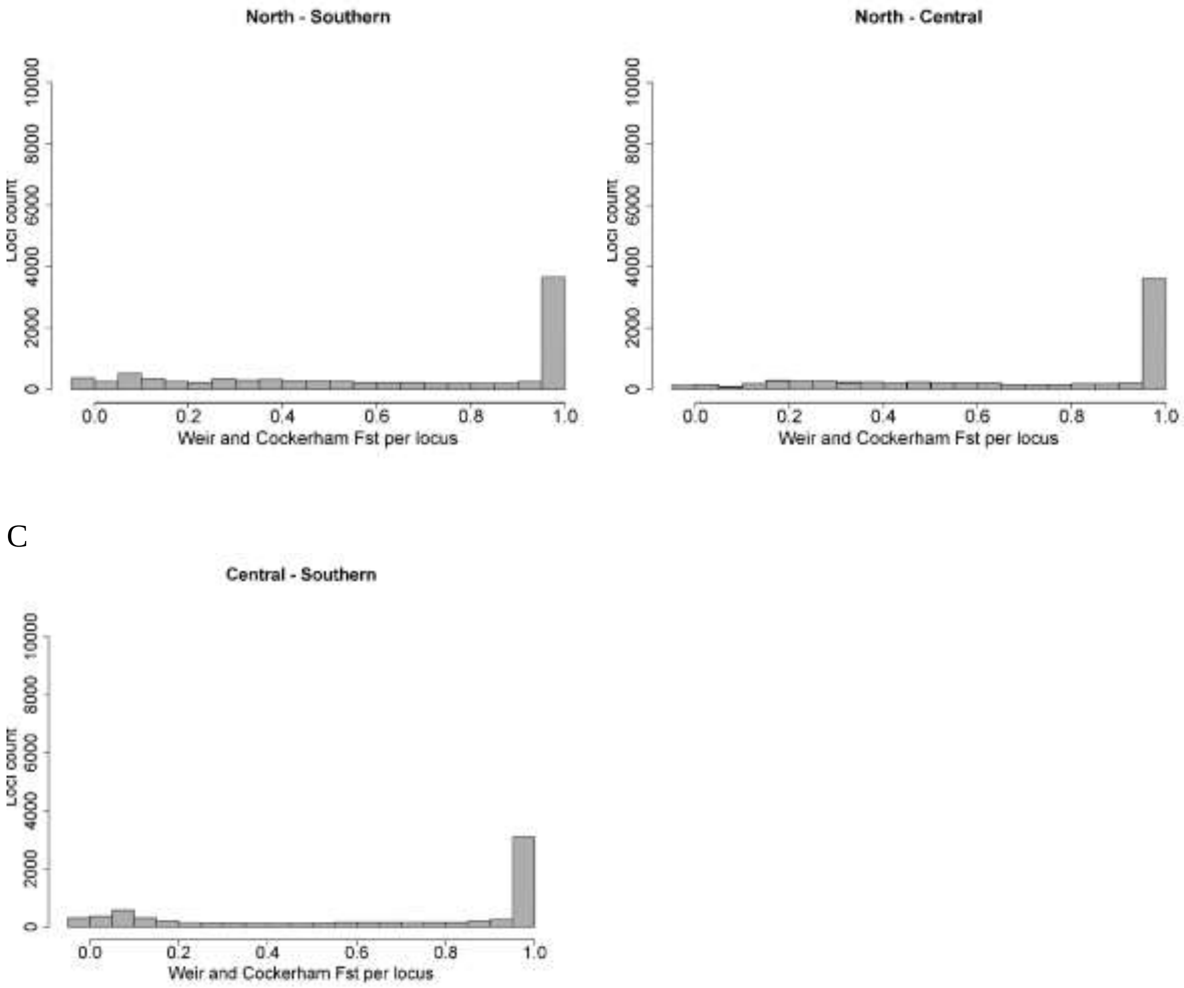

Fig. S25 Distribution of pairwise F_{ST} for all loci between clades (NZ-wide dataset, with all 10,987 loci). A) North-Central; B) North-Southern; C) Central-Southern.

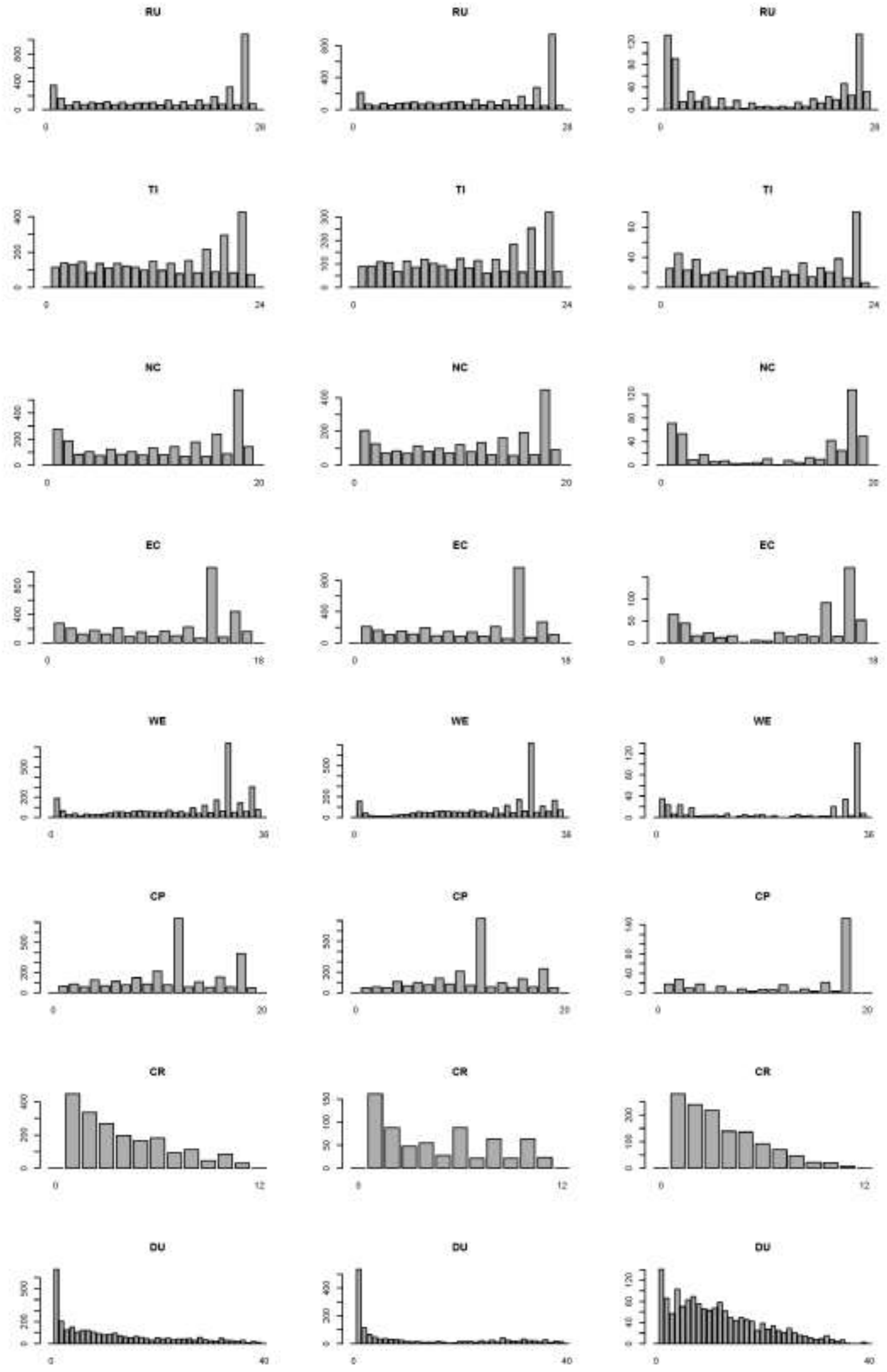

(figure continued in the next page)

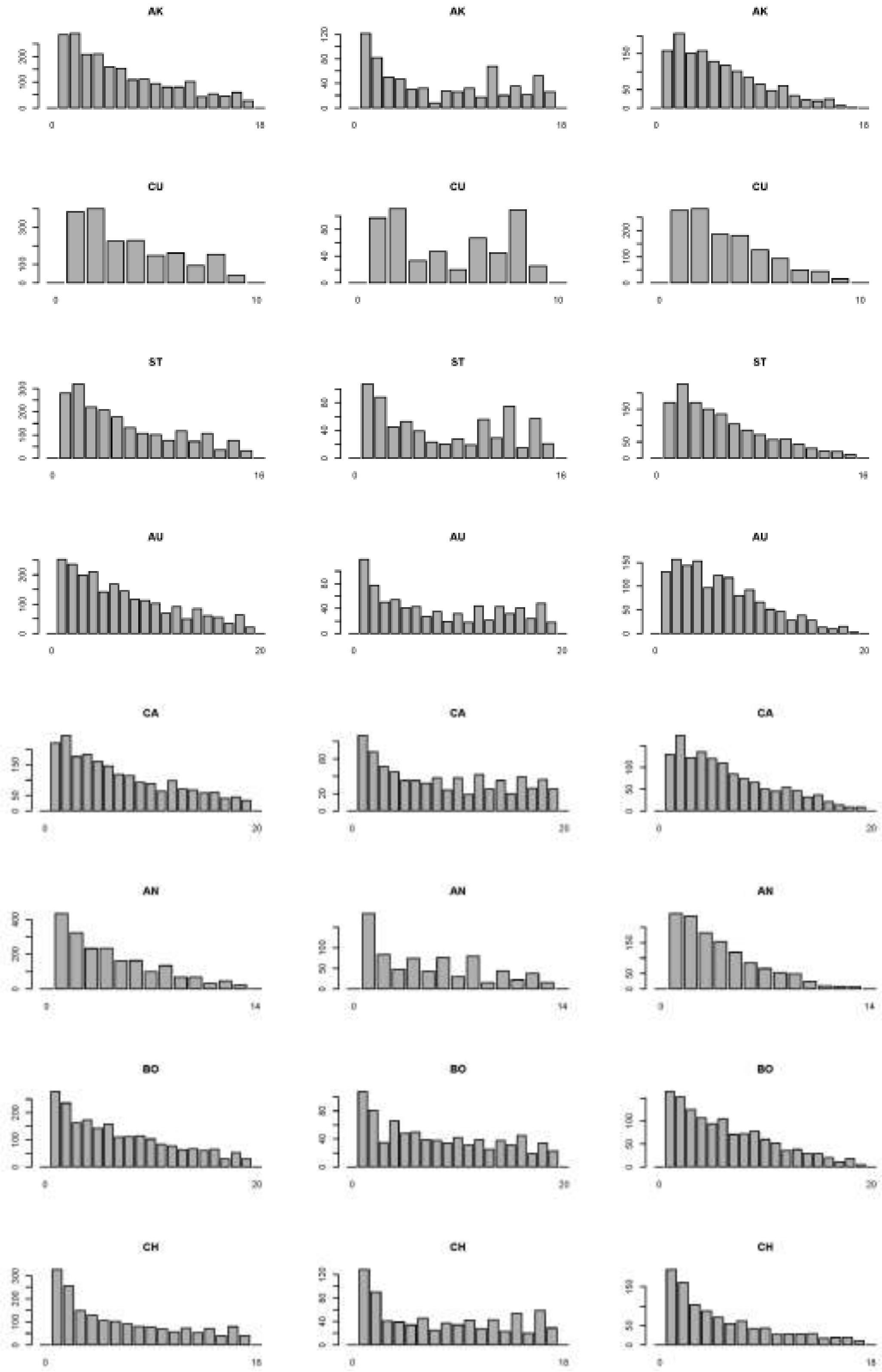

Fig. S26 Allele frequency spectrum per population, based on the NZ-wide dataset with all loci (left), remaining loci after removing combined ‘outlier’ markers (middle), and combined ‘outlier’ markers (right). Bayescan and PCAdapt results combined by the geometric mean of

q-values, cut-off = 0.05. Note that the population allele frequency spectra are based on counts from the NZ-wide datasets, and the larger number of South populations means the south alleles are the majority, and thus taken as reference.

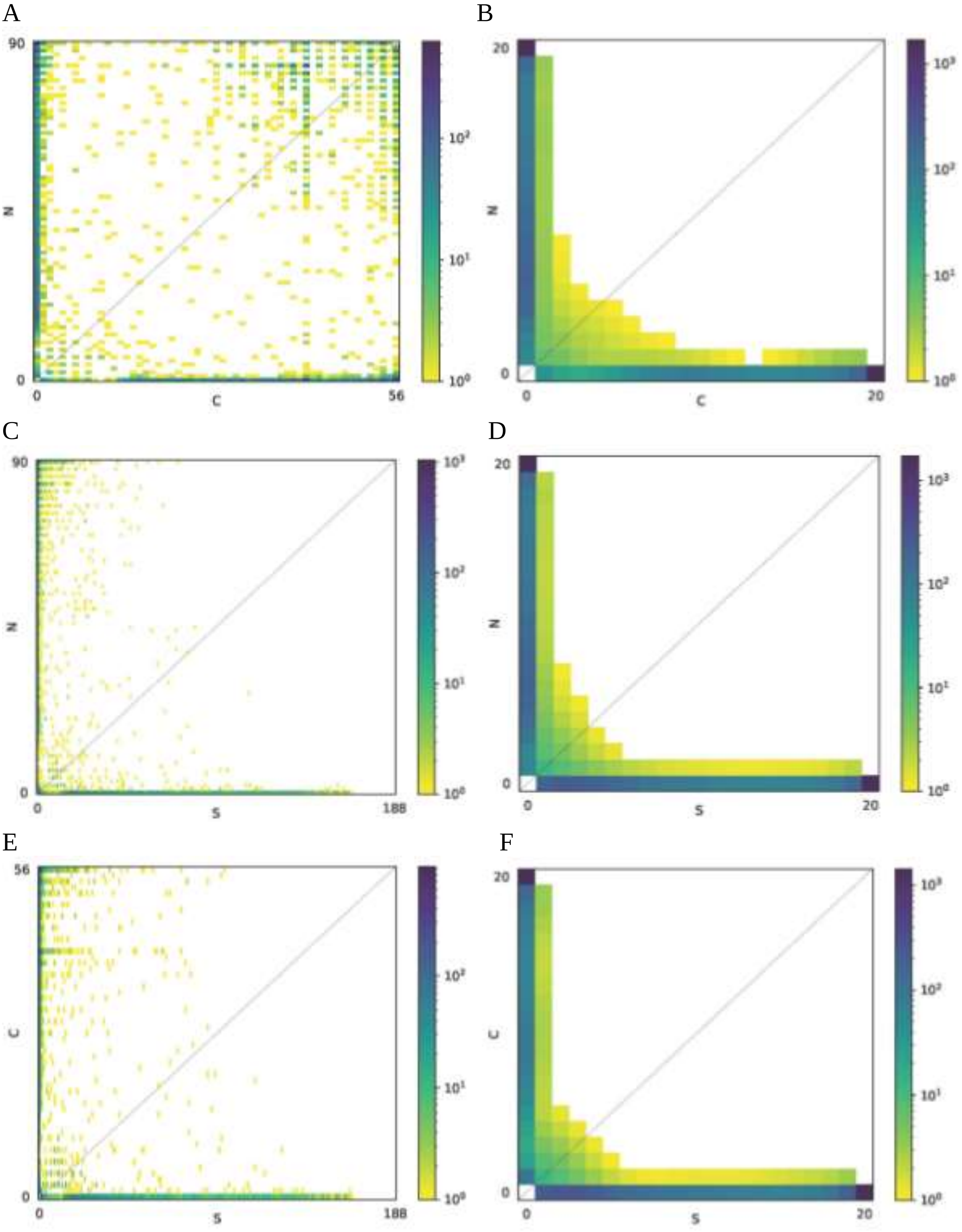

Fig. S27 2D allele frequency spectra comparing North-Central (A-B), North-South (C-D) and Central-South (E-F) clades, with all individuals per clade (left column) and with sample size projected down to 20 per clade (right column, as in Gutenkunst et al. 2009). Projecting

sample sizes down accounts for missing data and simplifes visualization. Allele frequency spectra estimated with the NZ-wide dataset (with all SNPs).

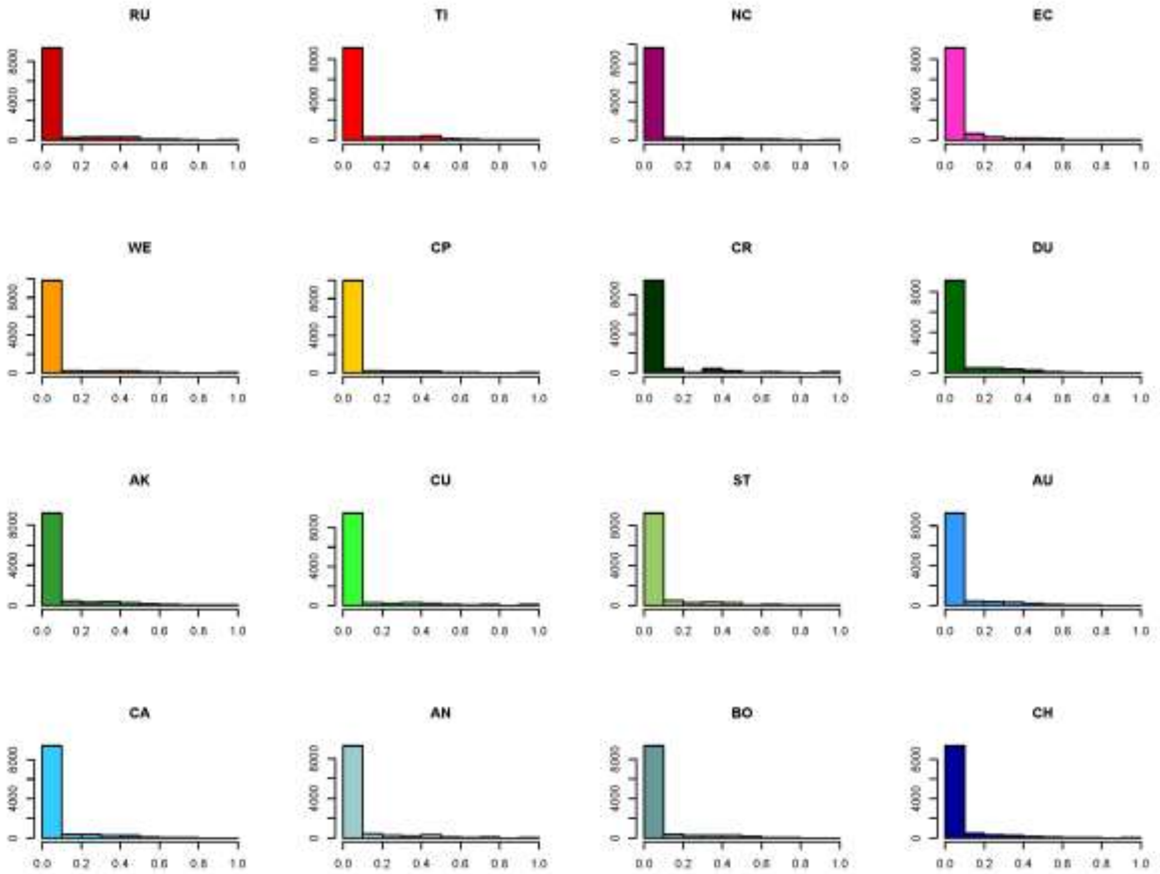

Fig. S28 Distribution of observed heterozygosity in all 10,987 loci NZ-wide, within each population.

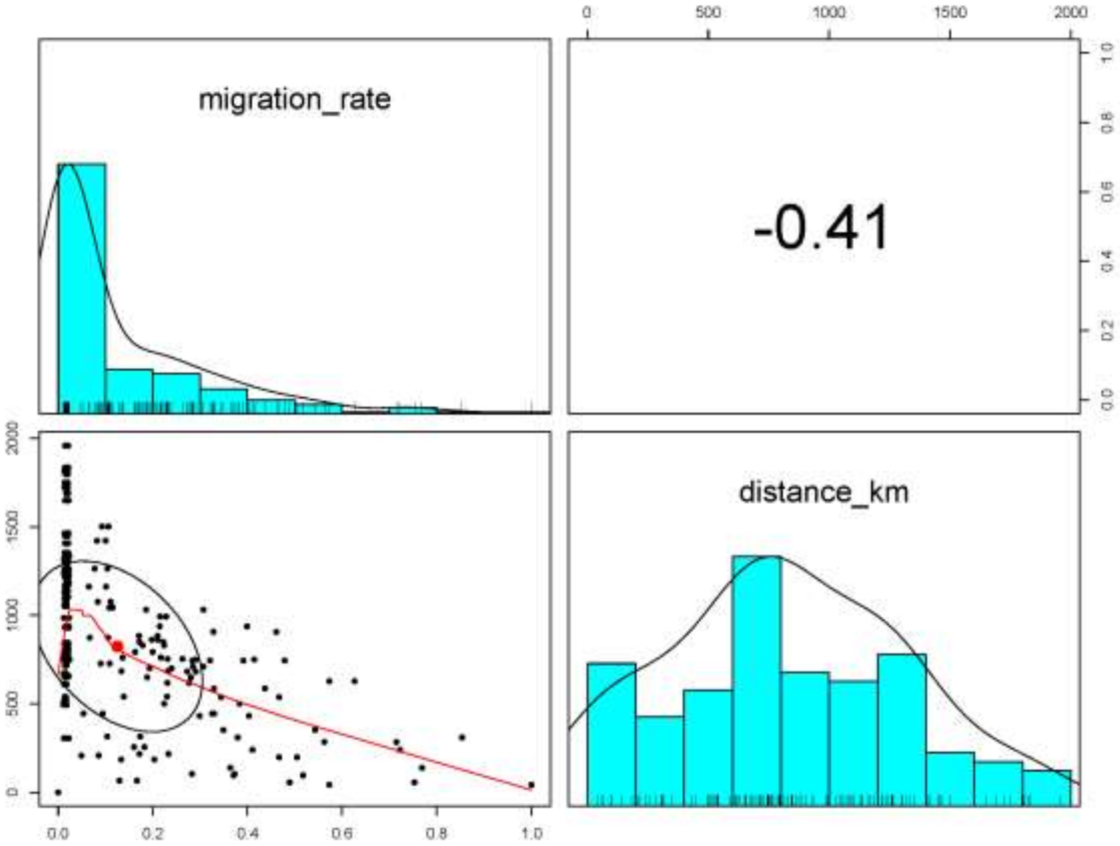

Fig. S29 Negative correlation of relative migration rate (estimated with the Gst method in the R package diveRsity) and shortest straight distance between populations (calculated using https://www.distance.to). R = -0.41, p < 0.001. Dataset: NZ-wide with all 10,987 loci.

A

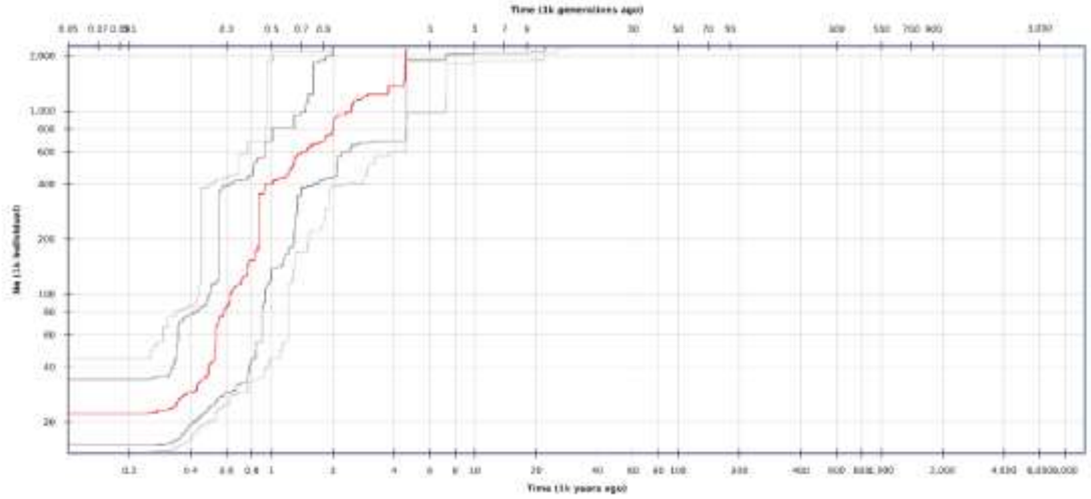

B

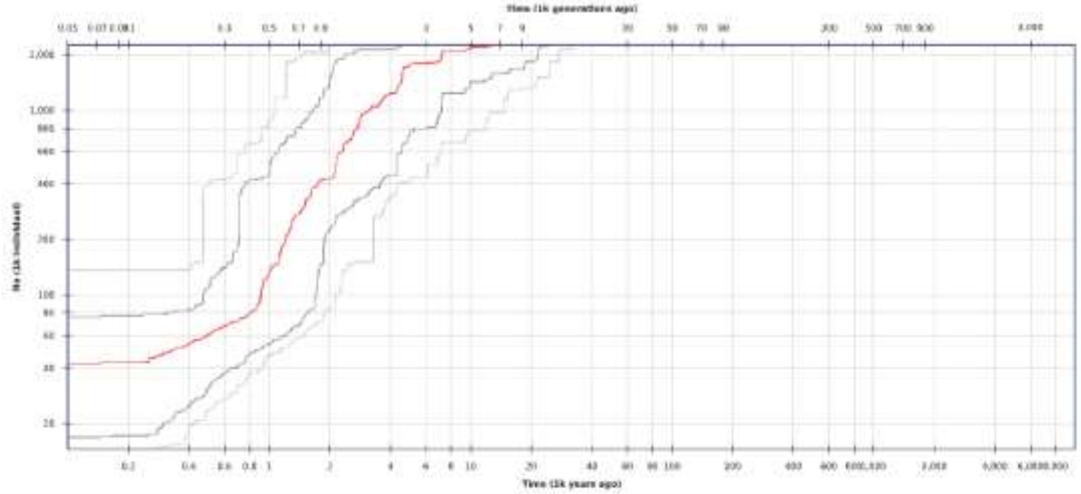

C

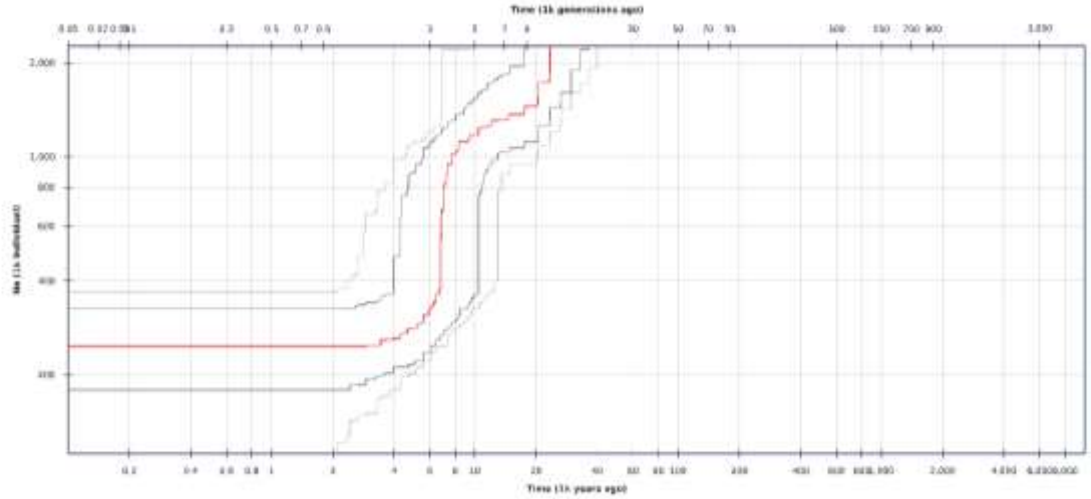

Fig. S30 Stairway plots estimated with the allele frequency spectra of the NZ-wide scale projected down to 20. Chiton generation times were set to 2 years (as in Ni et al. 2020); mutation rate was set to 1.1%/million year, or $2.2x10^{-8}$ (as in Salloum et al. 2020). A. NZ-

wide dataset with all 10,987 loci; B. NZ-wide remaining loci after removing combined ‘outlier’ markers; C. NZ-wide combined ‘outlier’ markers (Bayescan and PCAdapt results combined by the geometric mean of *q*-values, cut-off = 0.05). The top x-axis corresponds to number of generations, the bottom x-axis corresponds to million years, from present (left) to past (right). The y-axis corresponds to estimation of effective population size. Lighter grey lines correspond to upper and lower 12.5% confidence intervals, darker grey lines to 2.5% upper and lower confidence intervals, and red line to the median effective population size.

A. NZ-wide (remaining) loci vs. combined 'outlier' markers

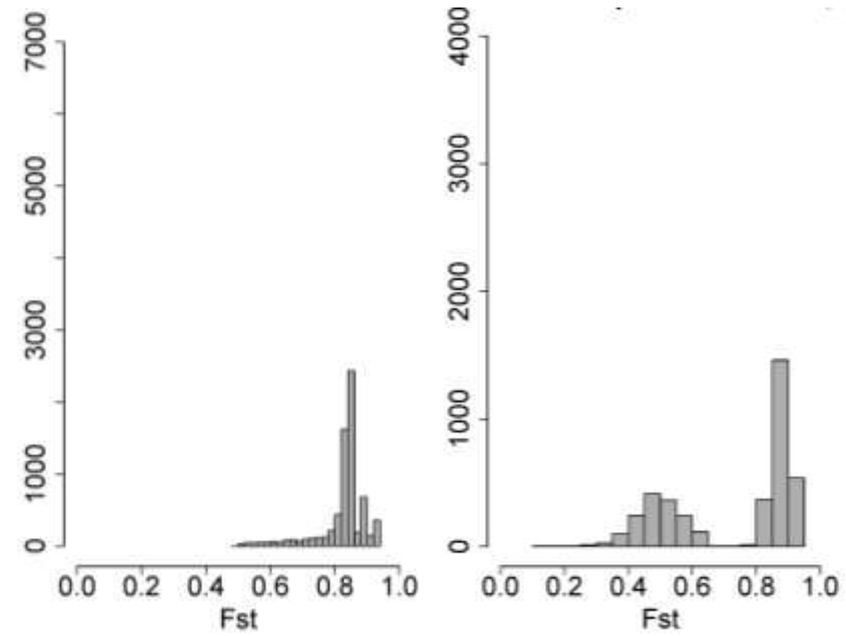

B. NI-specific (remaining) loci vs. combined 'outlier' markers

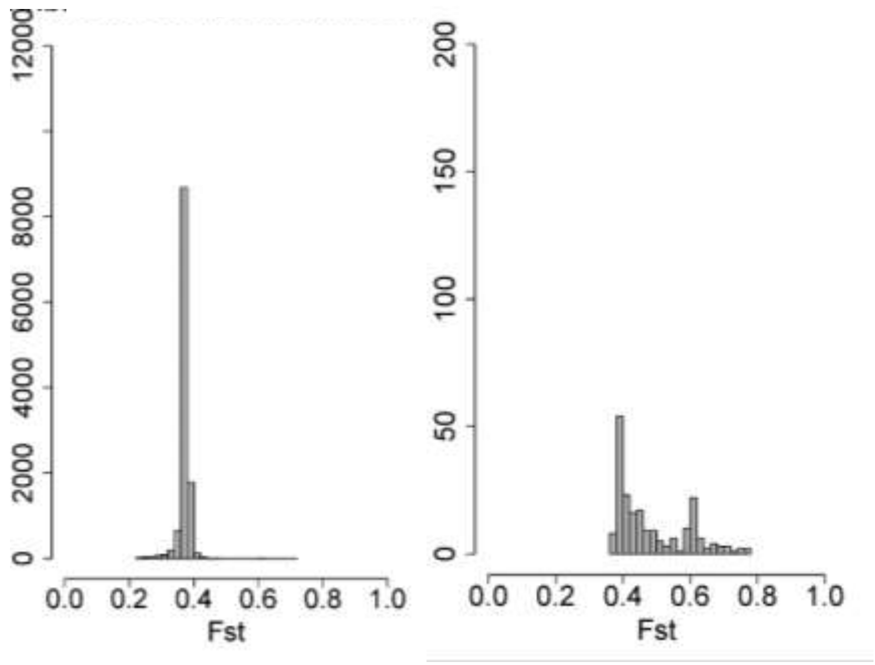

C. SI-specific (remaining) loci vs. combined ‘outlier’ markers

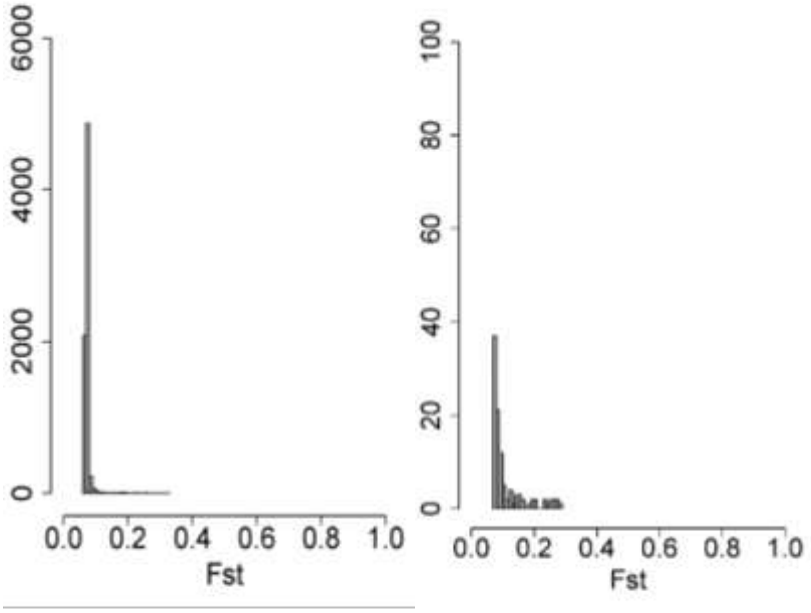

D. Southern (remaining) loci vs. combined ‘outlier’ markers

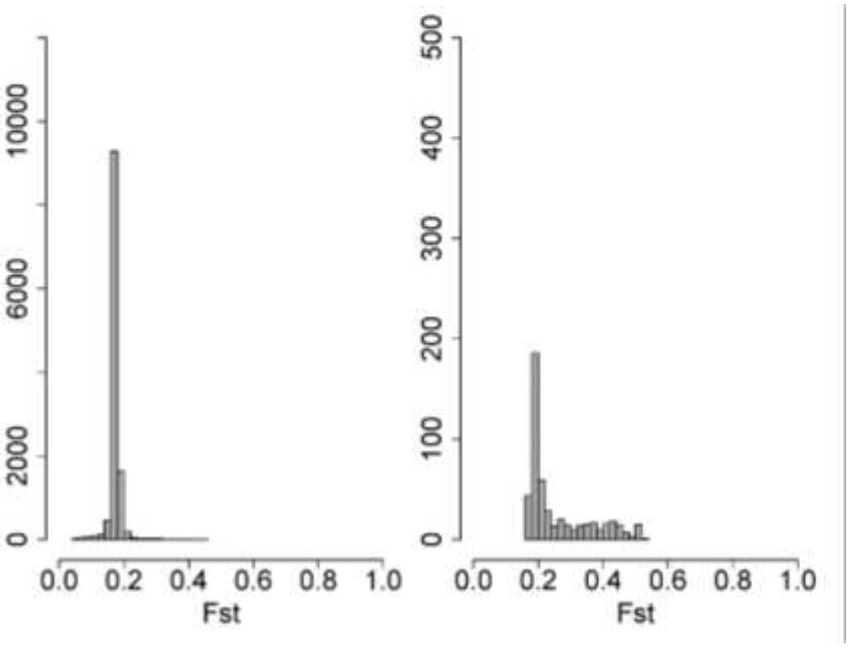

Fig. S31 Distribution of F_{ST} across loci, comparing remaining loci after removing combined ‘outlier’ markers (left) and combined ‘outlier’ markers (right) in each dataset. The count of loci (y-axis) corresponding to F_{ST} values (x-axis) is shown. A) NZ-wide; B) NI-specific; C) SI-specific; D) southern.

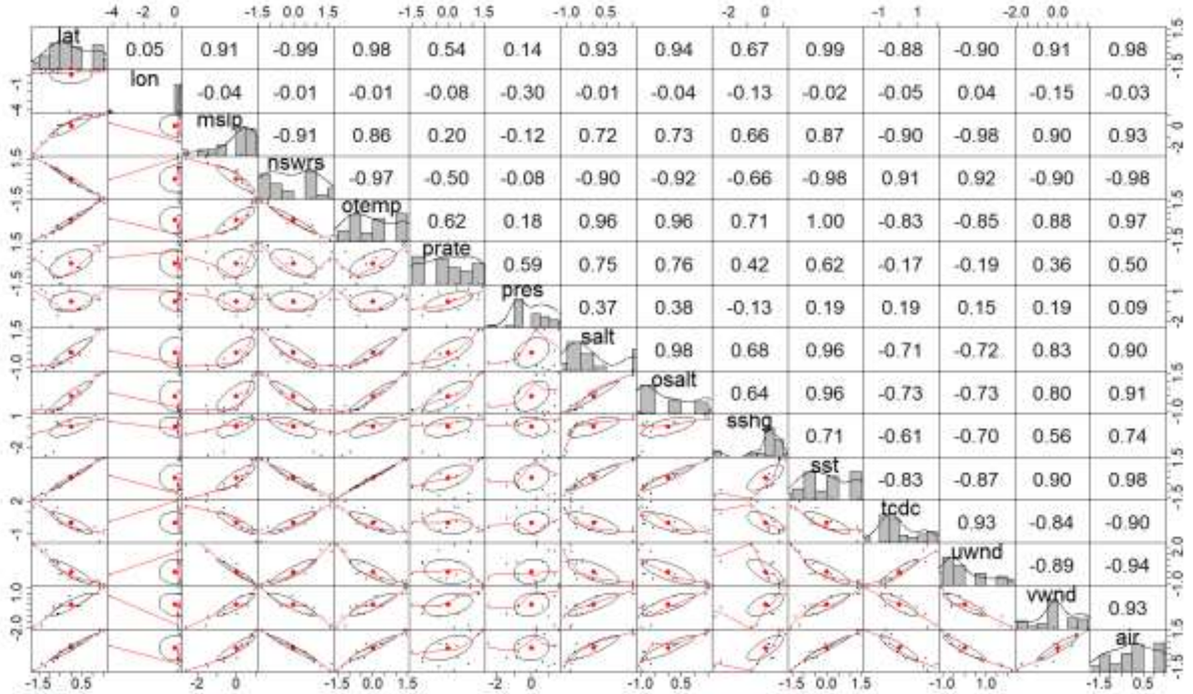

Fig. S32 Pairwise linear regressions and Pearson's correlation tests among environmental variables for the New Zealand-wide scale. Below diagonal are bivariate scatter plots, on the diagonal are histograms showing the distribution of the data, and above the diagonal are the Pearson's correlation coefficients. Environmental variables are latitude (lat), longitude (lon), mean sea level pressure (mslp), net shortwave (nswrs), ocean temperature (otemp), precipitation rate (prate), surface pressure (pres), salinity (salt), ocean salinity (osalt), sea surface height relative to geoid (sshg), sea surface temperature (sst), total cloud cover (tcdc), zonal wind velocity (uwnd), meridional wind velocity (vwnd), and air temperature (air).

A

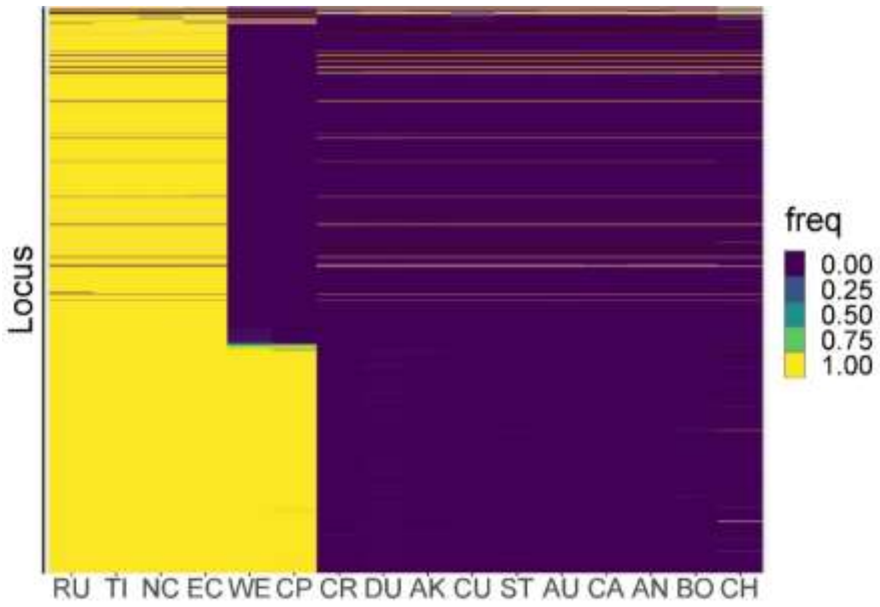

B

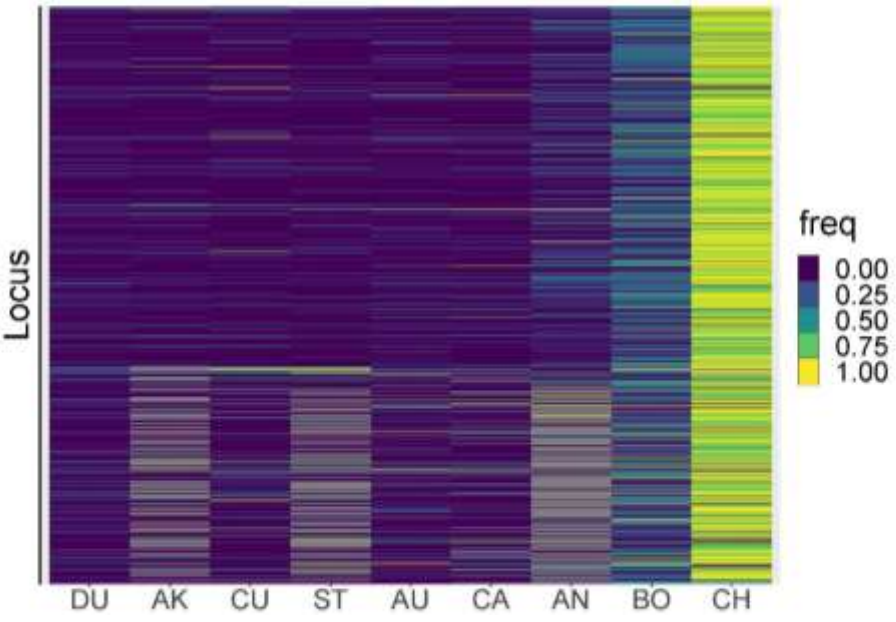

Fig. S33 Allele frequency of all GEA-loci (low-confidence and high-confidence results included). Allele frequencies are shown in a gradient of colours, blue corresponding to a frequency of 0 and yellow to a frequency of 1. Grey represents a missing locus in a population. A) NZ-wide 3,093 GEA-loci (detected with BayeScEnv, as RDA detected no associations); B) southern 743 GEA-loci (detected with RDA and BayeScEnv).

Supporting Tables

Table S1 Details on sampling size per population after SNP calling and filtering. Christchurch (CR) population was only included in the NZ-wide scale.

Clade	Population	Individuals sampled	Latitude	Longitude
North	RU	14	-35.24	174.12
	TI	12	-36.32	174.78
	NC	10	-36.70	175.61
	EC	9	-37.55	178.16
Central	WE	18	-41.33	174.83
	CP	10	-41.59	175.23
South	CR	6	-43.51	172.73
	DU	20	-45.95	170.34
	AK	9	-46.16	170.16
	CU	5	-46.66	169.10
	ST	8	-46.96	167.72
	AU	10	-50.63	166.14
	CA	10	-52.52	169.13
	AN	7	-49.69	178.77
	BO	10	-47.76	179.02
	CH	9	-43.95	-176.55

Table S2 Details on datasets, with a description on populations and SNPs within each dataset, and the analyses that were done using each dataset. No. pops is the number of populations included in each dataset; No. SNPs is the number of SNPs of each dataset (after filtering).

Dataset Name	No. Pops	Dataset Description	No. SNPs	Analyses
NZ-wide	16	16 populations in New Zealand and its Sub-Antarctic Islands	10,987	PCAdapt, Bayescan, RDA and BayeScEnv
North Island Subset	4	A subset of the NZ-wide dataset, including only the four populations of the North Clade (RU, TI, NC, EC)	3,076	PCAdapt and Bayescan
South Island Subset	5	A subset of the NZ-wide dataset, including only the five populations of the South Island Region (CR, DU, AK, CU, ST)	3,154	PCAdapt and Bayescan
Southern Subset	10	A subset of the NZ-wide dataset, including only the ten populations of the South Clade (CR, DU, AK, CU, ST, AU, CA, BO, AN, CH)	3,628	PCAdapt and Bayescan
North Island Specific (NI)	4	SNPs called specifically for the North Clade (resulting dataset includes four populations: RU, TI, NC, EC)	12,012	PCAdapt, Bayescan, RDA and BayeScEnv
South Island Specific (SI)	4	SNPs called specifically for South Island Region (resulting dataset includes four populations: DU, AK, CU, ST)	7,476	PCAdapt, Bayescan, RDA and BayeScEnv
Southern Specific	9	SNPs called specifically for South Clade (resulting dataset includes nine populations: DU, AK, CU, ST, AU, CA, BO, AN, CH)	13,003	PCAdapt, Bayescan, RDA and BayeScEnv

Table S3 Pairwise matrix of shortest straight geographic distance (in kilometres) between populations.

	WE	TI	ST	RU	NC	EC	DU	CU	CR	CP	CH	CA	BO	AU	AN	AK
WE	0.00															
TI	493.65	0.00														
ST	842.46	1,265.22	0.00													
RU	672.59	185.69	1,406.80	0.00												
NC	507.21	65.99	1,302.24	207.60	0.00											
EC	511.50	315.30	1353.71	445.14	256.01	0.00										
DU	615.30	1,064.63	239.94	1,219.05	1095.53	1126.17	0.00									
CU	750.17	1,187.24	103.62	1,335.29	1,221.27	1,261.62	138.76	0.00								
CR	305.59	765.18	538.21	928.89	791.49	816.98	310.04	444.64	0.00							
CP	56.24	531.97	842.59	713.82	540.05	518.53	610.08	747.19	304.83	0.00						
CH	774.49	1,085.03	1,263.74	1,259.23	1,051.64	845.42	1043.07	1162.37	873.78	720.22	0.00					
CA	1,319.14	1,796.55	618.30	1,958.33	1,820.96	1809.63	744.15	649.76	1031.41	1297.82	1421.52	0.00				
BO	794.52	1,262.28	848.24	1,447.67	1,256.45	1,138.42	682.80	761.08	684.38	744.26	540.86	884.31	0.00			
AU	1,241.52	1,688.34	431.69	1,835.25	1,721.80	1,750.33	628.00	501.12	937.46	1231.97	1,501.48	284.34	992.92	0.00		
AN	985.36	1,464.61	862.11	1,649.14	1462.35	1353.13	749.82	792.75	830.05	938.38	727.36	745.33	216.86	906.64	0.00	
AK	657.69	1,104.14	198.53	1,256.89	1135.86	1,168.71	42.83	96.32	352.25	652.85	1076.68	710.25	700.81	586.68	753.60	0.00

Table S4 Number of loci associated with each environmental variable, split by low and high confidence, method, and spatial scale. 'NZ' is the NZ-wide scale; 'S' is southern clade scale. 'Combined' is the result of combining *q*-values by the geometric mean of results for both methods. $^{+}$ indicates a common locus between two environmental variables after combining the *q*-values. Key of environmental variables: latitude (lat), longitude (lon), and monthly long term means of sea level pressure (mslp), net shortwave (nswrs), ocean temperature (otemp), precipitation rate (prate), surface pressure (press), salinity (salt), ocean salinity (osalt), sea surface height relative to geoid (sshg), sea surface temperature (sst), total cloud cover (tcdc), zonal wind velocity (uwnd), meridional wind velocity (vwnd), and air temperature (air). Note that only six variables were tested in the RDA, after excluding other variables that were highly correlated with latitude.

**Loci**	**Low-confidence**				**High-confidence**	
**Method**	**RDA**		**BayeScEnv**		**Combined**	
**Scale**	**NZ**	**S**	**NZ**	**S**	**NZ**	**S**
Lat	0	0	0	72	0	81
Mslp*	.	.	0	82	.	.
Nswrs*	.	.	2,704	75	.	.
Otemp*	.	.	485	19	.	.
Salt*	.	.	1,252	6	.	.
Osalt*	.	.	3,064	17	.	.
Sst*	.	.	0	32	.	.
Tcdc*	.	.	0	101	.	.
Uwnd*	.	.	2,872	64	.	.
Air*	.	.	12	13	.	.
Vwnd*	.	.	0	15	.	.
Lon	0	3	0	13	0	4^{+}
Prate	0	705	0	46	0	1
Press	0	0	0	44	0	0
Sshg	0	2	0	11	0	1^{+}
Total	0	708	3,093	135	0	86

*correlation of >0.7 with latitude, hence not analysed in RDA

Table S5 Number of loci associated with each environmental variable, split by low and high confidence, method, and spatial scale. ‘NI’ is the North Island (specific) scale; ‘SI’ is South Island (specific) clade scale. ‘Combined’ is the result of combining *q*-values by the geometric mean of results for both methods. Key of environmental variables: latitude (lat), longitude (lon), and monthly long term means of sea level pressure (mslp), net shortwave (nswrs), ocean temperature (otemp), precipitation rate (prate), surface pressure (press), salinity (salt), ocean salinity (osalt), sea surface height relative to geoid (sshg), sea surface temperature (sst), total cloud cover (tcdc), zonal wind velocity (uwnd), meridional wind velocity (vwnd), and air temperature (air). Note that only six variables were tested in the RDA, after excluding other variables that were highly correlated with latitude.

**Loci**	**Low-confidence**				**High-confidence**	
**Method**	**RDA**		**BayeScEnv**		**Combined**	
**Scale**	**NI**	**SI**	**NI**	**SI**	**NI**	**SI**
Lat	0	0	2	0	0	81
Mslp*	.	.	8	0	.	.
Nswrs*	.	.	8	0	.	.
Otemp*	.	.	6	1	.	.
Salt*	.	.	7	0	.	.
Osalt*	.	.	6	4	.	.
Sst*	.	.	8	5	.	.
Tcdc*	.	.	6	1	.	.
Uwnd*	.	.	7	0	.	.
Air*	.	.	8	0	.	.
Vwnd*	.	.	8	0	.	.
Lon	0	0	6	1	0	0
Prate***	0	0	8	1	0	0
Press**	0	0	8	0	0	0
Sshg	0	0	6	0	0	0
Total	0	0	15	10	0	0

*correlation of >0.7 with latitude, hence not analysed in RDA; **correlation of >0.7 with latitude in the NI scale only, hence not analysed in the NI RDA; ***correlation of >0.7 with latitude in the SI scale only, hence not analysed in the SI RDA;

Table S6 Average heterozygosity (observed) across loci per population within each dataset for all loci (A), remaining loci after removing combined 'outlier' loci (R), and combined 'outlier' markers (O), after combining the geometric mean of *q*-values between PCAdapt and Bayescan.

	**NZ-wide**			**NI**			**SI**			**southern**		
	A	R	O	A	R	O	A	R	O	A	R	O
**RU**	0.063	0.087	0.021	0.210	0.215	0.066						
**TI**	0.070	0.090	0.035	0.221	0.222	0.152						
**NC**	0.056	0.080	0.014	0.186	0.187	0.105						
**EC**	0.056	0.078	0.019	0.171	0.172	0.054						
**WE**	0.045	0.067	0.008									
**CP**	0.041	0.059	0.008									
**CR**	0.057	0.018	0.125									
**DU**	0.061	0.024	0.125				0.253	0.252	0.246	0.211	0.223	0.037
**AK**	0.061	0.020	0.133				0.258	0.259	0.098	0.225	0.239	0.033
**CU**	0.060	0.018	0.134				0.258	0.258	0.120	0.216	0.229	0.035
**ST**	0.060	0.019	0.129				0.249	0.250	0.077	0.206	0.217	0.039
**AU**	0.060	0.024	0.124							0.222	0.233	0.057
**CA**	0.055	0.021	0.114							0.211	0.222	0.043
**AN**	0.059	0.023	0.120							0.225	0.231	0.131
**BO**	0.061	0.031	0.112							0.234	0.231	0.253
**CH**	0.045	0.024	0.080							0.172	0.168	0.206

Table S7 Pairwise matrix of relative migration rates (method Gst). Note that rates are of asymmetric migration (not the same above and below diagonal, as it depends on the source population).

Population	AK	AN	AU	BO	CA	CH	CP	CR	CU	DU	EC	NC	RU	ST	TI	WE
AK	-	0.23	0.44	0.24	0.31	0.11	0.02	0.35	0.37	1.00	0.02	0.02	0.02	0.47	0.02	0.02
AN	0.26	-	0.46	0.23	0.32	0.11	0.02	0.18	0.16	0.41	0.02	0.02	0.02	0.21	0.02	0.02
AU	0.33	0.33	-	0.23	0.56	0.11	0.02	0.21	0.22	0.63	0.02	0.02	0.02	0.30	0.02	0.02
BO	0.19	0.17	0.22	-	0.17	0.14	0.02	0.13	0.14	0.27	0.02	0.02	0.02	0.17	0.02	0.02
CA	0.28	0.28	0.71	0.21	-	0.10	0.02	0.19	0.19	0.48	0.02	0.02	0.02	0.23	0.02	0.02
CH	0.08	0.09	0.09	0.23	0.08	-	0.02	0.07	0.06	0.11	0.02	0.02	0.02	0.08	0.02	0.02
CP	0.02	0.02	0.02	0.02	0.02	0.02	-	0.02	0.02	0.02	0.02	0.02	0.02	0.02	0.02	0.75
CR	0.54	0.22	0.40	0.23	0.31	0.11	0.02	-	0.33	0.85	0.02	0.02	0.02	0.47	0.02	0.02
CU	0.52	0.20	0.38	0.22	0.28	0.10	0.02	0.33	-	0.77	0.02	0.02	0.02	0.37	0.02	0.02
DU	0.57	0.29	0.57	0.29	0.39	0.12	0.01	0.38	0.36	-	0.01	0.01	0.01	0.41	0.01	0.01
EC	0.01	0.01	0.01	0.01	0.01	0.01	0.01	0.01	0.01	0.01	-	0.16	0.09	0.01	0.17	0.01
NC	0.01	0.01	0.01	0.01	0.01	0.01	0.01	0.01	0.01	0.01	0.18	-	0.09	0.01	0.17	0.01
RU	0.01	0.01	0.01	0.01	0.01	0.01	0.02	0.01	0.01	0.02	0.05	0.05	-	0.01	0.20	0.02
ST	0.50	0.20	0.40	0.22	0.28	0.10	0.02	0.34	0.28	0.72	0.02	0.02	0.02	-	0.02	0.02
TI	0.02	0.02	0.02	0.02	0.02	0.02	0.02	0.02	0.02	0.02	0.10	0.13	0.13	0.02	-	0.02
WE	0.01	0.01	0.01	0.01	0.01	0.01	0.49	0.01	0.01	0.01	0.01	0.01	0.01	0.01	0.01	-

Table S8 Blast results for the high-confidence GEA loci (query) to the *A. granulata* genome and transcriptome (subject). Out of the 86 high-confidence loci, 11 were included in the Blast search (loci with high heterozygosity and missing data across populations were excluded) Query id is the identifier for the *O. neglectus* locus; subject id is the name of the genomic region that was hit in the *A. granulata* genome. q.start corresponds to the starting base pair of the match in the query sequence; q.end corresponds to the last base pair of the match in the query sequence; s.start corresponds to the genomic position where the match starts for the subject sequence; s.end corresponds to the genomic position with the last base pair of the match in the subject sequence; *A. granuata* database indicates what sequence was used from *A. granulata* (Scaffolds or either of the available transcriptomes); Only hits to the transcriptome had the flanking subject sequence extracted and blasted against the mollusc taxid ('Blasted *A. gran* flanking sequence?' column).

query id	subject id	alignment length	query length	subject length	q. start	q. end	s. start	s. end	e-value	*A. granulata* Database	Blasted *A. gran* flanking sequence?
CLocus_14183	Scaffold_100010	29	92	22611271	23	51	1245313	1245341	1.41E-06	Scaffolds	No
CLocus_46591	Scaffold_100024	90	92	6064231	1	90	3754823	3754912	8.34E-19	Scaffolds	No
CLocus_46591	model.g24769.t1	90	92	1029	1	90	918	829	1.30E-19	80Ktranscripts	Yes
CLocus_46591	model.g24769.t1	90	92	1029	1	90	918	829	4.74E-20	20000transcripts	Yes

References

de Villemereuil, P., Frichot, E., Bazin, E., Francois, O. & Gaggiotti, O.E. (2014) Genome scan methods against more complex models: when and how much should we trust them? *Molecular Ecology,* **23,** 2006-2019. 10.1111/mec.12705

Gutenkunst, R.N., Hernandez, R.D., Williamson, S.H. & Bustamante, C.D. (2009) Inferring the joint demographic history of multiple populations from multidimensional SNP frequency data. *PLoS Genet,* **5,** e1000695. 10.1371/journal.pgen.1000695

Ni, G., Kim, T., Shin, Y., Park, J., Lee, Y., Kil, H.J. & Park, J.K. (2020) Life-history features and oceanography drive phylogeographic patterns of the chiton Acanthochitona cf. rubrolineata (Lischke, 1873) in the northwestern Pacific. *PeerJ,* **8,** e8794. 10.7717/peerj.8794

Salloum, P.M., De Villemereuil, P., Santure, A.W., Waters, J.M. & Lavery, S.D. (2020) Hitchhiking consequences for genetic and morphological patterns: the influence of kelp-rafting on a brooding chiton. *Biological Journal of the Linnean Society,* **130,** 756-777. 10.1093/biolinnean/blaa073/5859172
